# Anticholinergic Drug Exposure and the Risk of Dementia: A Nested Case-Control Study

**DOI:** 10.1001/jamainternmed.2019.0677

**Published:** 2019-06-24

**Authors:** Carol A. C. Coupland, Trevor Hill, Tom Dening, Richard Morriss, Michael Moore, Julia Hippisley-Cox

**Affiliations:** 1 Division of Primary Care, University of Nottingham, Nottingham, England; 2 Division of Psychiatry and Applied Psychology, Institute of Mental Health, Nottingham, England; 3 University of Southampton Medical School, Primary Care and Population Sciences, Aldermoor Health Centre, Southampton, England; 4 Nuffield Department of Primary Care Health Sciences, University of Oxford, Oxford, England

## Abstract

**Importance:**

Anticholinergic medicines have short-term cognitive adverse effects, but it is uncertain whether long-term use of these drugs is associated with an increased risk of dementia.

**Objective:**

To assess associations between anticholinergic drug treatments and risk of dementia in persons 55 years or older.

**Design, Setting, and Participants:**

This nested case-control study took place in general practices in England that contributed to the QResearch primary care database. The study evaluated whether exposure to anticholinergic drugs was associated with dementia risk in 58 769 patients with a diagnosis of dementia and 225 574 controls 55 years or older matched by age, sex, general practice, and calendar time. Information on prescriptions for 56 drugs with strong anticholinergic properties was used to calculate measures of cumulative anticholinergic drug exposure. Data were analyzed from May 2016 to June 2018.

**Exposures:**

The primary exposure was the total standardized daily doses (TSDDs) of anticholinergic drugs prescribed in the 1 to 11 years prior to the date of diagnosis of dementia or equivalent date in matched controls (index date).

**Main Outcomes and Measures:**

Odds ratios (ORs) for dementia associated with cumulative exposure to anticholinergic drugs, adjusted for confounding variables.

**Results:**

Of the entire study population (284 343 case patients and matched controls), 179 365 (63.1%) were women, and the mean (SD) age of the entire population was 82.2 (6.8) years. The adjusted OR for dementia increased from 1.06 (95% CI, 1.03-1.09) in the lowest overall anticholinergic exposure category (total exposure of 1-90 TSDDs) to 1.49 (95% CI, 1.44-1.54) in the highest category (>1095 TSDDs), compared with no anticholinergic drug prescriptions in the 1 to 11 years before the index date. There were significant increases in dementia risk for the anticholinergic antidepressants (adjusted OR [AOR], 1.29; 95% CI, 1.24-1.34), antiparkinson drugs (AOR, 1.52; 95% CI, 1.16-2.00), antipsychotics (AOR, 1.70; 95% CI, 1.53-1.90), bladder antimuscarinic drugs (AOR, 1.65; 95% CI, 1.56-1.75), and antiepileptic drugs (AOR, 1.39; 95% CI, 1.22-1.57) all for more than 1095 TSDDs. Results were similar when exposures were restricted to exposure windows of 3 to 13 years (AOR, 1.46; 95% CI, 1.41-1.52) and 5 to 20 years (AOR, 1.44; 95% CI, 1.32-1.57) before the index date for more than 1095 TSDDs. Associations were stronger in cases diagnosed before the age of 80 years. The population-attributable fraction associated with total anticholinergic drug exposure during the 1 to 11 years before diagnosis was 10.3%.

**Conclusions and Relevance:**

Exposure to several types of strong anticholinergic drugs is associated with an increased risk of dementia. These findings highlight the importance of reducing exposure to anticholinergic drugs in middle-aged and older people.

## Key Points

**Question:**

Is the risk of dementia among persons 55 years or older associated with the use of different types of anticholinergic medication?

**Findings:**

In this nested case-control study of 58 769 patients with a diagnosis of dementia and 225 574 matched controls, there were statistically significant associations of dementia risk with exposure to anticholinergic antidepressants, antiparkinson drugs, antipsychotic drugs, bladder antimuscarinics, and antiepileptic drugs after adjusting for confounding variables.

**Meaning:**

The associations observed for specific types of anticholinergic medication suggest that these drugs should be prescribed with caution in middle-aged and older adults.

## Teaser

This nested case-control study including 58 769 patients diagnosed with dementia and 225 574 matched controls 55 years or older evaluates whether, and to what extent, anticholinergic drug exposure was associated with dementia risk.

## Introduction

An estimated 47 million people worldwide were living with dementia in 2015,^1^ while in the United States around 5.7 million people have Alzheimer dementia.^2^ Modifiable risk factors, including hypertension, hearing loss, depression, diabetes, and smoking, account for around 35% of dementia cases.^1^ Anticholinergic drugs are another potentially modifiable risk factor. This broad group of drugs acts by blocking the neurotransmitter acetylcholine in the central and peripheral nervous system and includes some antihistamines, antidepressants, and medications for gastrointestinal and bladder disorders. These medicines can have short-term adverse effects, including confusion and memory loss in older people,^3,4,5,6^ but it is less certain whether long-term use increases the risk of dementia.

Observational studies of anticholinergic drugs and dementia risk^7,8,9,10^ have generally been relatively small, only assessed short-term exposure, or were subject to recall bias. These studies were also susceptible to protopathic bias because they did not account for anticholinergic drugs being prescribed to treat early symptoms of dementia before diagnosis. A cohort study^11^ that reduced protopathic bias by excluding prescriptions in the final year of follow-up found that higher cumulative anticholinergic drug use was associated with a significantly increased risk of dementia but had limited power for analysis of separate types of anticholinergic drug. A recent larger study^12^ found varying risks associated with different types of anticholinergic drugs and concluded that further research should examine individual anticholinergic drug classes.

This study was designed to assess the association between cumulative anticholinergic drug use and risk of dementia in a large, representative British population. The study objectives were to estimate dementia risks associated with different types of anticholinergic medication including analyses of prescriptions up to 20 years before diagnosis.

## Methods

### Study Design

This was a nested case-control study within a cohort of patients registered with practices in England contributing to the QResearch database (version 41). QResearch is an anonymized research database of more than 30 million individuals in over 1500 general practices that includes data recorded prospectively from routine health care. The data include demographic information, medical diagnoses, prescriptions, referrals, laboratory results, and clinical values.

The study was approved in accordance with the agreed procedure with the East Midlands Derby Research Ethics Committee, waiving written informed consent for deidentified patient data.

### Selection of Cases and Controls

The base cohort included patients 55 years and older registered during the study period (January 1, 2004, to January 31, 2016) without a diagnosis of dementia at study entry, defined as the latest of the study start date (January 1, 2004), the patient’s 55th birthday, date of registration with the practice plus 1 year, or date when the practice computer system was installed plus 1 year. The cohort were followed up until the earliest date of death, transfer to another practice, or the study end date (January 31, 2016).

Case patients were those diagnosed with dementia during follow-up, identified using clinical codes recorded in the practice records or linked Office of National Statistics death records. Patients with prescriptions for acetylcholinesterase-inhibiting drugs (donepezil, galantamine, memantine, and rivastigmine) but without a recorded diagnosis of dementia were also included because these drugs are licensed only for patients with dementia. Case patients with diagnostic codes for specific subtypes of dementia associated with Huntington disease, Parkinson disease, Creutzfeldt-Jakob disease, or human immunodeficiency virus (HIV) were excluded, as were patients diagnosed with Parkinson disease, Huntington disease, or multiple sclerosis to reduce indication bias.

Each case patient was matched to 5 controls by age (within 1 year), sex, general practice, and calendar time using incidence density sampling.^13^ The index date for controls was the date of diagnosis for their matched case patient. Controls were excluded if they had a diagnosis of Parkinson disease, Huntington disease, or multiple sclerosis.

For the primary analyses, case patients and controls were only included if they had at least 11 years of recorded data prior to the index date, so that anticholinergic drug exposure could be assessed over a complete period of 10 years (excluding the 1-year period prior to the index date).

### Exposures

There is incomplete consensus on which drugs are considered as having anticholinergic properties. We used the approach of Gray et al,^11^ which included drugs identified as having strong anticholinergic properties by the American Geriatrics Society 2012 Beers Criteria Update Expert Panel.^14^ We also included additional drugs in the Beers 2015 updated list of strong anticholinergic drugs,^15^ drugs listed as having a high anticholinergic burden in the Anticholinergic Cognitive Burden scale,^16^ or identified as high-potency anticholinergics in a systematic review,^17^ and some further drugs identified as having substantial anticholinergic properties in the British National Formulary; these may have been omitted in previous studies owing to their unavailability in the country where the study originated. eTable 1 in the Supplement shows the 56 anticholinergic drugs included in the study with details of their basis for inclusion.

We extracted details of prescriptions for the included anticholinergic drugs. To reduce protopathic biases, we did not include prescriptions issued in the year before the index date. In 2 additional analyses, we only included prescriptions issued up to 3 and up to 5 years before diagnosis.

The primary exposure variable was total cumulative anticholinergic drug exposure, which combined the different types of anticholinergic medications based on the method used by Gray et al.^11^ This involved calculating the total dose of each prescription by multiplying the number of tablets prescribed by the dose per tablet (or equivalent for solutions, inhalers, injections, or patches). These values were then divided by minimum effective daily dose values recommended for use in older adults to give a number of standardized daily doses for each prescription. We used minimum effective dose values from the Geriatric Dosage Handbook^18^ where available, and for the additional drugs we used the lowest recommended dose values (in older people if stated) in the British National Formulary (see eTable 1 in the Supplement). We summed these standardized values over all anticholinergic prescriptions in the exposure time windows of interest to obtain total standardized daily doses (TSDDs) for each patient.

We also calculated TSDDs for each type of anticholinergic drug based on its main indication (antihistamines, antidepressants, antivertigo/antiemetic drugs, antiparkinson agents, antipsychotics, bladder antimuscarinics, skeletal muscle relaxants, gastrointestinal antispasmodics, antiarrhythmics, antiepileptic drugs, and antimuscarinic bronchodilators). As a sensitivity analysis we used World Health Organization (WHO)-defined daily dose (DDD) values (https://www.whocc.no/atc_ddd_index/) to standardize the prescribed doses.

### Confounding Variables

We accounted for potential confounding variables identified as risk factors for dementia or indications for anticholinergic drug use,^19,20,21,22,23,24,25^ including body mass index, calculated as weight in kilograms divided by height in meters squared,^20^ smoking status,^26^ alcohol consumption,^27^ Townsend deprivation score,^21^ self-assigned ethnic group,^28^ comorbidities (coronary heart disease, atrial fibrillation, heart failure, hypertension, hyperlipidemia, diabetes, stroke, subarachnoid hemorrhage, transient ischemic attack, renal failure, asthma, chronic obstructive pulmonary disease, anxiety, bipolar disorder, depression, Down syndrome, severe learning difficulties, schizophrenia, severe head injury, and cognitive decline/memory loss), and use of other medications (antihypertensive drugs, aspirin, hypnotic and anxiolytic drugs, nonsteroidal antiinflammatory drugs, statins). These variables were evaluated at the start of the exposure window for the primary analysis.

### Statistical Analysis

We used conditional logistic regression to estimate odds ratios (ORs) adjusted for the confounding variables. The exposure window in the main analyses comprised the 1 to 11 years before the index date. We categorized the anticholinergic exposure variable into 5 categories (0, 1-90, 91-365, 366-1095, and >1095 TSDDs).^11^ Similarly we assessed associations for the 11 separate types of anticholinergic drug. Data were analyzed from May 2016 to June 2018.

We carried out subgroup analyses and interaction tests by age at index date (younger than 80 years and 80 years and older), by sex, and separately in case patients diagnosed with Alzheimer disease (including mixed), vascular dementia, and other or unspecified types of dementia with their respective matched controls.

We carried out the following sensitivity analyses:

(1) we assessed anticholinergic exposure over a time window of 3 to 13 years before the index date by excluding prescriptions in the 3 years before the index date;
(2) we assessed anticholinergic exposure over a time window of 5 to 20 years before the index date to further reduce potential protopathic biases and to assess associations for longer term exposure;
(3) we removed those anticholinergic drugs not included by Gray et al^11^ so we could directly compare associations;
(4) we used multiple imputation by chained equations to replace missing values for body mass index, smoking status, and alcohol consumption. We created 10 multiply imputed data sets and combined results using Rubin rules^29^; and
(5) we repeated the analyses using the cumulative exposure variable standardized by WHO DDD values.

We calculated population-attributable fractions by combining adjusted odds ratios (AORs) with the proportions of cases in the different categories of anticholinergic drug exposure.^30,31^ We used *P* < .01 (2-tailed) to determine statistical significance. We used Stata (version 15.1) for all analyses.

## Results

The base cohort comprised 3 638 582 individuals aged 55 to 100 years. During a total of 20 005 739 person-years of follow-up, 128 517 people were diagnosed with dementia. After applying exclusion criteria, 58 769 case patients and 225 574 matched controls were eligible for inclusion (eFigure 1 in the Supplement). Case patients had a mean (SD) age of 82.4 (7.0) years at diagnosis, and 63.1% (37 105) were women (Table 1); eTable 2 in the Supplement details that in the 36 666 cases where dementia type was recorded, 22 034 (60.1%) patients had a diagnosis of Alzheimer disease (including mixed), 13 313 (36.3%) had a diagnosis of vascular dementia, and 1319 (3.6%) had other types of dementia.

**Table 1.** Demographic Characteristics of Case Patients and Matched Controls

Characteristic	Study Participants, No. (%)	
	Case Patients (n = 58 769)	Controls (n = 225 574)
Age at diagnosis of dementia/index date, mean (SD), y	82.4 (7.0)	82.1 (6.8)
Age at diagnosis/index date, y		
55-64	685 (1.2)	2577 (1.1)
65-74	6983 (11.9)	26 952 (12.0)
75-84	26 610 (45.3)	106 705 (47.3)
85-94	23 075 (39.3)	86 148 (38.2)
≥95	1416 (2.4)	3192 (1.4)
Men	21 664 (36.9)	83 314 (36.9)
Women	37 105 (63.1)	142 260 (63.1)
Ethnicity recorded	45 008 (76.6)	174 660 (77.4)
Ethnicity		
White/not recorded	57 004 (97.0)	220 081 (97.6)
Indian	341 (0.6)	1302 (0.6)
Pakistani	138 (0.2)	431 (0.2)
Bangladeshi	107 (0.2)	271 (0.1)
Other Asian	110 (0.2)	444 (0.2)
Caribbean	629 (1.1)	1726 (0.8)
Black African	99 (0.2)	292 (0.1)
Chinese	43 (0.1)	217 (0.1)
Other	298 (0.5)	810 (0.4)
Townsend deprivation score, fifths^a^		
1 (least deprived)	15 802 (26.9)	65 507 (29.0)
2	14 628 (24.9)	57 835 (25.6)
3	13 003 (22.1)	48 432 (21.5)
4	9558 (16.3)	34 310 (15.2)
5 (most deprived)	5701 (9.7)	19 244 (8.5)
BMI recorded	53 518 (91.1)	204 764 (90.8)
BMI, mean (SD)	26.5 (4.4)	26.8 (4.4)
Smoking status		
Nonsmoker	33 936 (57.7)	132 732 (58.8)
Ex-smoker	16 285 (27.7)	61 168 (27.1)
Light smoker (1-9 cigarettes/d)	4455 (7.6)	15 795 (7.0)
Moderate smoker (10-19 cigarettes/d)	1795 (3.1)	6443 (2.9)
Heavy smoker (≥20 cigarettes/d)	1069 (1.8)	3714 (1.7)
Not recorded	1229 (2.1)	5722 (2.5)
Alcohol intake		
Nondrinker	20 193 (34.4)	73 287 (32.5)
Trivial (<1 U/d)	19 253 (32.8)	75 330 (33.4)
Light (1-2 U/d)	6928 (11.8)	27 494 (12.2)
Moderate (3-6 U/d)	6117 (10.4)	24 443 (10.8)
Heavy (7-9 U/d)	468 (0.8)	1510 (0.7)
Very heavy (>9 U/d)	124 (0.2)	356 (0.2)
Not recorded	5686 (9.7)	23 154 (10.3)
Missing values for BMI, smoking status, or alcohol intake	7770 (13.2)	30 758 (13.6)

Abbreviations: BMI, body mass index, calculated as weight in kilograms divided by height in meters squared; U/d, units of alcohol per day.

^a^ Deprivation fifths using quintiles for the entire QResearch database across all ages.

Table 2 presents information on comorbidities and prescribed medications. Prevalence values were slightly higher in case patients than in controls for all the comorbidities and prescribed medications.

**Table 2.** Prevalence of Comorbidities and Prescribed Medications in Case Patients and Matched Controls

Characteristic	Study Participants, No. (%)	
	Case Patients (n = 58 769)	Controls (n = 225 574)
Comorbidity^a^		
Hypertension	19 907 (33.9)	73 267 (32.5)
Stroke	1483 (2.5)	3936 (1.7)
Transient ischemic attack	1717 (2.9)	5110 (2.3)
Subarachnoid hemorrhage	177 (0.3)	453 (0.2)
Coronary heart disease	7778 (13.2)	25 872 (11.5)
Heart failure	990 (1.7)	3149 (1.4)
Atrial fibrillation	1774 (3.0)	5900 (2.6)
Hyperlipidemia	4123 (7.0)	13 986 (6.2)
Diabetes	4612 (7.9)	12 929 (5.7)
Anxiety	3693 (6.3)	12 348 (5.5)
Depression	8106 (13.8)	26 086 (11.6)
Bipolar disorder	152 (0.3)	262 (0.1)
Schizophrenia	265 (0.5)	559 (0.3)
Severe head injury	211 (0.4)	689 (0.3)
Severe learning difficulties	6 (0.0)	5 (0.0)
Down syndrome	54 (0.1)	2 (0.0)
Cognitive decline	184 (0.3)	364 (0.2)
Asthma	4482 (7.6)	16 243 (7.2)
Chronic obstructive pulmonary disease	1558 (2.7)	5228 (2.3)
Renal disease	176 (0.3)	594 (0.3)
Medications^a^		
Aspirin	12 162 (20.7)	40 329 (17.9)
Nonsteroidal antiinflammatory drugs	21 222 (36.1)	79 412 (35.2)
Antihypertensives	25 377 (43.2)	92 708 (41.1)
Statins	7804 (13.3)	25 218 (11.2)
Anxiolytic	2756 (4.7)	8822 (3.9)
Hypnotic	4749 (8.1)	15 855 (7.0)

^a^ Comorbidities and medications assessed at index date minus 11 years.

### Anticholinergic Drug Exposure

In the 1 to 11 years before the index date, 56.6% of case patients (33 253) and 51.0% of controls (115 096) were prescribed at least 1 anticholinergic drug, with a median of 6 prescriptions in case patients and 4 in controls (Table 3). The most frequently prescribed types of anticholinergic drugs were antidepressants (27.1% of case patients, 23.3% of controls), antivertigo/antiemetic drugs (23.8% of case patients, 21.7% of controls), and bladder antimuscarinic drugs (11.7% of case patients, 8.3% of controls) (see eFigure 2 in the Supplement). eTable 3 in the Supplement provides descriptive information for the 56 different anticholinergic drugs included in the study. eTables 4 and 5 in the Supplement present descriptive information on anticholinergic drugs prescribed in the 3 to 13 years and 5 to 20 years before the index date, respectively.

**Table 3.** Numbers of Case Patients and Controls Prescribed Different Types of Anticholinergic Drugs in the 1 to 11 Years Before the Index Date

Anticholinergic Drug Group	Case Patients (n = 58 769)				Controls (n = 225 574)				
	No. (%)		Median (IQR)			No. (%)		Median (IQR)	
	No. With Prescriptions	Total Prescriptions	No. of Prescriptions^a^	Total Dose^a^^,^^b^		No. With Prescriptions	Total Prescriptions	No. of Prescriptions^a^	Total Dose^a^^,^^b^
Any anticholinergic drugs	33 253 (56.6)	952 263 (100)	6 (2-34)	214 (42-1531)		115 096 (51.0)	2 504 790 (100)	4 (1-22)	136 (30-982)
Antihistamines	6457 (11.0)	34 151 (3.6)	1 (1-3)	30 (23-84)		23 145 (10.3)	117 271 (4.7)	1 (1-3)	30 (27-84)
Antidepressants	15 938 (27.1)	427 489 (44.9)	6 (1-35)	280 (62-1876)		52 560 (23.3)	1 141 284 (45.6)	4 (1-25)	196 (56-1350)
Antivertigo/antiemetic drugs	13 969 (23.8)	79 673 (8.4)	2 (1-4)	20 (9-56)		48 990 (21.7)	249 214 (9.9)	1 (1-3)	19 (9-50)
Antiparkinson drugs	292 (0.5)	16 498 (1.7)	31 (3-91)	879 (105-3274)		527 (0.2)	25 412 (1.0)	22 (2-73)	541 (48-2333)
Antipsychotic drugs	1812 (3.1)	69 895 (7.3)	11 (2-51)	756 (119-3751)		3400 (1.5)	109 180 (4.4)	8 (1-46)	490 (84-2894)
Bladder antimuscarinic drugs	6864 (11.7)	170 064 (17.9)	8 (2-32)	330 (60-1461)		18 778 (8.3)	362 677 (14.5)	5 (1-23)	198 (56-1120)
Skeletal muscle relaxants	429 (0.7)	1361 (0.1)	1 (1-2)	23 (16-45)		1568 (0.7)	5202 (0.2)	1 (1-2)	24 (17-42)
Gastrointestinal antispasmodic drugs	4036 (6.9)	29 320 (3.1)	1 (1-4)	30 (13-120)		15 481 (6.9)	101 268 (4.0)	1 (1-3)	28 (13-112)
Antiarrhythmic drugs	49 (0.1)	2569 (0.3)	31 (5-88)	882 (175-2345)		172 (0.1)	8142 (0.3)	37 (5-77)	1148 (150-2436)
Antiepileptic drugs	1411 (2.4)	41 360 (4.3)	4 (1-39)	153 (42-2240)		4492 (2.0)	97 180 (3.9)	2 (1-20)	80 (30-970)
Antimuscarinic bronchodilator drugs	3878 (6.6)	79 883 (8.4)	8 (2-29)	300 (60-1330)		13 996 (6.2)	287 960 (11.5)	8 (2-29)	330 (67-1333)

Abbreviations: IQR, interquartile range; TSDD, total standardized daily dose.

^a ^ In patients with 1 or more prescriptions for drug.

^b ^ Cumulative dose calculated using TSDDs in exposure window.

### Associations With Dementia

The AOR associated with total cumulative anticholinergic exposure in the 1 to 11 years before the index date increased from 1.06 (95% CI, 1.03-1.09) for 1 to 90 TSDDs to 1.49 (95% CI, 1.44-1.54) for more than 1095 TSDDs, compared with nonuse (Table 4). Results were similar but with slightly lower ORs when restricted to the 3 to 13 and 5 to 20 years before the index date; for example, for the 5 to 20 years before the index date the AOR was 1.44 (95% CI, 1.32-1.57) for more than 1095 TSDDs (Table 4).

**Table 4.** Risk of Dementia Associated With Total Cumulative Use of Any Type of Anticholinergic Drugs Among Study Patients

Exposure Category	Study Participants, No (%)		OR (95% CI)	
	Case Patients	Controls	Unadjusted	Adjusted^a^
**Exposure in the 1 to 11 Years Before Index Date**				
Patients, No.	58 769	225 574	NA	NA
Cumulative use (TSDDs)				
Nonuse	25 516 (43.4)	110 478 (49.0)	1 [Reference]	1 [Reference]
1-90	12 546 (21.4)	50 220 (22.3)	1.09 (1.06-1.11)	1.06 (1.03-1.09)
91-365	6370 (10.8)	23 302 (10.3)	1.20 (1.16-1.24)	1.17 (1.13-1.21)
366-1095	4537 (7.7)	14 138 (6.3)	1.40 (1.35-1.46)	1.36 (1.30-1.41)
>1095	9800 (16.7)	27 436 (12.2)	1.58 (1.53-1.62)	1.49 (1.44-1.54)
**Exposure in the 3 to 13 Years Before Index Date**				
Patients, No.	45 621	169 020	NA	NA
Cumulative use (TSDDs)				
Nonuse	20 545 (45.0)	84 676 (50.1)	1 [Reference]	1 [Reference]
1-90	9749 (21.4)	37 553 (22.2)	1.07 (1.05-1.10)	1.05 (1.02-1.08)
91-365	5079 (11.1)	17 470 (10.3)	1.22 (1.17-1.26)	1.18 (1.13-1.23)
366-1095	3286 (7.2)	10 423 (6.2)	1.32 (1.27-1.38)	1.25 (1.19-1.31)
>1095	6962 (15.3)	18 898 (11.2)	1.55 (1.50-1.60)	1.46 (1.41-1.52)
**Exposure in the 5 to 20 Years Before Index Date**				
Patients, No.	8283	27 200	NA	NA
Cumulative use (TSDDs)				
Nonuse	3335 (40.3)	12 281 (45.2)	1 [Reference]	1 [Reference]
1-90	1924 (23.2)	6531 (24.0)	1.09 (1.02-1.16)	1.07 (1.00-1.15)
91-365	1003 (12.1)	3278 (12.1)	1.15 (1.06-1.25)	1.11 (1.01-1.21)
366-1095	699 (8.4)	1811 (6.7)	1.44 (1.31-1.59)	1.33 (1.20-1.48)
>1095	1322 (16.0)	3299 (12.1)	1.52 (1.40-1.64)	1.44 (1.32-1.57)

Abbreviations: OR, odds ratio; NA, not applicable; TSDD, total standardized daily dose.

^a ^ Adjusted for body mass index, calculated as weight in kilograms divided by height in meters squared, smoking status, alcohol consumption, Townsend deprivation score, ethnic group, coronary heart disease, atrial fibrillation, heart failure, hypertension, hyperlipidemia, diabetes (type 1 and type 2), stroke, transient ischemic attack, subarachnoid hemorrhage, renal disease, asthma, chronic obstructive pulmonary disease, anxiety, depression, bipolar disorder, schizophrenia, severe head injury, cognitive decline/memory loss, antihypertensive drugs, aspirin, hypnotics, anxiolytic drugs, nonsteroidal antiinflammatory drugs, statins, and with matching by age, sex, general practice, and calendar time.

Among specific types of anticholinergic drugs there were significant increases in risk associated with use of antidepressants, antiparkinson drugs, antipsychotics, bladder antimuscarinics, and antiepileptic drugs (Table 5). Adjusted odds ratios in the highest exposure category (>1095 TSDDs) were 1.29 (95% CI, 1.24-1.34) for antidepressants, 1.52 (95% CI, 1.16-2.00) for antiparkinson drugs, 1.70 (95% CI, 1.53-1.90) for antipsychotics, 1.65 (95% CI, 1.56-1.75) for bladder antimuscarinics, and 1.39 (95% CI, 1.22-1.57) for antiepileptic drugs, all compared with nonuse. For antivertigo/antiemetic drugs, as detailed in Table 5, there was a significantly increased risk associated with 366 to 1095 TSDDs, but not for the highest exposure category. There were no significant increases in risk associated with antihistamines, skeletal muscle relaxants, gastrointestinal antispasmodics, antiarrhythmics, or antimuscarinic bronchodilators, although the numbers of patients exposed were small for skeletal muscle relaxants and antiarrhythmics. Patterns of risk were similar in the 3- to 13- and 5- to 20-year exposure windows (eTable 6 in the Supplement), except for antipsychotic drug exposure in the 5- to 20-year window, where there were no statistically significant increases in risk; the AOR for more than 1095 TSDDs was 1.23 (95% CI, 0.93-1.62). For some drug types, numbers were too small to allow analysis for the 5 to 20 years before the index date (eTable 7 in the Supplement).

**Table 5.** ORs for Total Cumulative Use of Different Types of Anticholinergic Drugs in the 1 to 11 Years Before the Index Date

Drug Type	Study Participants, No. (%)		Odds Ratio (95%CI)		
	Case Patients (n = 58 769)	Controls (n = 225 574)	Unadjusted	Adjusted for the Other Drug Types^a^	Fully Adjusted^b^
**Antihistamines, TSDDs**					
Nonuse	52 312 (89.0)	202 429 (89.7)	1 [Reference]	1 [Reference]	1 [Reference]
1-90	4987 (8.5)	18 187 (8.1)	1.05 (1.02-1.09)	1.02 (0.98-1.05)	1.03 (0.99-1.07)
91-365	923 (1.6)	3105 (1.4)	1.14 (1.06-1.23)	1.06 (0.99-1.15)	1.03 (0.95-1.12)
366-1095	280 (0.5)	1022 (0.5)	1.06 (0.93-1.22)	0.98 (0.85-1.12)	1.02 (0.88-1.18)
>1095	267 (0.5)	831 (0.4)	1.22 (1.06-1.41)	1.14 (0.99-1.31)	1.14 (0.98-1.34)
**Antidepressants, TSDDs**					
Nonuse	42 831 (72.9)	173 014 (76.7)	1 [Reference]	1 [Reference]	1 [Reference]
1-90	5098 (8.7)	19 402 (8.6)	1.08 (1.04-1.11)	1.04 (1.01-1.08)	1.02 (0.98-1.06)
91-365	3463 (5.9)	11 931 (5.3)	1.20 (1.15-1.24)	1.14 (1.10-1.19)	1.12 (1.07-1.17)
366-1095	2227 (3.8)	6749 (3.0)	1.35 (1.29-1.42)	1.27 (1.20-1.33)	1.25 (1.18-1.32)
>1095	5150 (8.8)	14 478 (6.4)	1.47 (1.42-1.52)	1.34 (1.29-1.39)	1.29 (1.24-1.34)
**Antivertigo/Antiemetics, TSDDs**					
Nonuse	44 800 (76.2)	176 584 (78.3)	1 [Reference]	1 [Reference]	1 [Reference]
1-90	11 427 (19.4)	41 159 (18.3)	1.10 (1.07-1.12)	1.06 (1.03-1.08)	1.05 (1.02-1.08)
91-365	1574 (2.7)	5026 (2.2)	1.23 (1.16-1.31)	1.14 (1.08-1.21)	1.14 (1.07-1.21)
366-1095	617 (1.1)	1659 (0.7)	1.47 (1.34-1.61)	1.33 (1.21-1.47)	1.41 (1.27-1.56)
>1095	351 (0.6)	1146 (0.5)	1.20 (1.06-1.35)	1.06 (0.94-1.20)	1.08 (0.94-1.24)
**Antiparkinson Agents, TSDDs**					
Nonuse	58 477 (99.5)	225 047 (99.8)	1 [Reference]	1 [Reference]	1 [Reference]
1-90	68 (0.1)	179 (0.1)	1.43 (1.08-1.90)	1.04 (0.78-1.38)	1.01 (0.73-1.39)
91-365	50 (0.1)	59 (0)	3.29 (2.25-4.81)	2.07 (1.40-3.05)	1.68 (1.09-2.58)
366-1095	39 (0.1)	71 (0)	2.08 (1.40-3.09)	1.29 (0.86-1.94)	1.03 (0.66-1.61)
>1095	135 (0.2)	218 (0.1)	2.39 (1.93-2.97)	1.61 (1.29-2.03)	1.52 (1.16-2.00)
**Antipsychotics, TSDDs**					
Nonuse	56 957 (96.9)	222 174 (98.5)	1 [Reference]	1 [Reference]	1 [Reference]
1-90	388 (0.7)	882 (0.4)	1.71 (1.51-1.93)	1.56 (1.38-1.76)	1.44 (1.25-1.66)
91-365	332 (0.6)	695 (0.3)	1.90 (1.66-2.17)	1.67 (1.46-1.91)	1.41 (1.21-1.65)
366-1095	304 (0.5)	490 (0.2)	2.45 (2.12-2.83)	2.15 (1.85-2.49)	2.09 (1.76-2.47)
>1095	788 (1.3)	1333 (0.6)	2.29 (2.09-2.50)	1.89 (1.72-2.07)	1.70 (1.53-1.90)
**Bladder Antimuscarinics, TSDDs**					
Nonuse	51 905 (88.3)	206 796 (91.7)	1 [Reference]	1 [Reference]	1 [Reference]
1-90	2139 (3.6)	7005 (3.1)	1.21 (1.15-1.27)	1.18 (1.12-1.24)	1.19 (1.13-1.26)
91-365	1417 (2.4)	4078 (1.8)	1.38 (1.30-1.47)	1.33 (1.25-1.41)	1.35 (1.27-1.45)
366-1095	1244 (2.1)	2941 (1.3)	1.71 (1.59-1.83)	1.63 (1.52-1.74)	1.65 (1.53-1.78)
>1095	2064 (3.5)	4754 (2.1)	1.73 (1.64-1.82)	1.65 (1.57-1.74)	1.65 (1.56-1.75)
**Skeletal Muscle Relaxants, TSDDs**					
Nonuse	58 340 (99.3)	224 006 (99.3)	1 [Reference]	1 [Reference]	1 [Reference]
1-90	372 (0.6)	1380 (0.6)	1.08 (0.96-1.22)	1.01 (0.89-1.14)	0.98 (0.86-1.11)
91-365	39 (0.1)	115 (0.1)	1.38 (0.95-1.99)	1.17 (0.81-1.70)	1.12 (0.77-1.65)
366-1095	9 (0)	41 (0)	0.90 (0.44-1.88)	0.84 (0.40-1.75)	0.99 (0.46-2.10)
>1095	9 (0)	32 (0)	1.09 (0.52-2.29)	0.90 (0.42-1.91)	1.10 (0.47-2.55)
**Gastrointestinal Antispasmodics, TSDDs**					
Nonuse	54 733 (93.1)	210 093 (93.1)	1 [Reference]	1 [Reference]	1 [Reference]
1-90	2765 (4.7)	10 914 (4.8)	0.97 (0.93-1.01)	0.90 (0.86-0.94)	0.90 (0.85-0.94)
91-365	722 (1.2)	2686 (1.2)	1.05 (0.96-1.14)	0.94 (0.86-1.02)	0.93 (0.85-1.02)
366-1095	267 (0.5)	938 (0.4)	1.11 (0.97-1.27)	0.98 (0.85-1.12)	0.93 (0.80-1.09)
>1095	282 (0.5)	943 (0.4)	1.19 (1.04-1.36)	1.03 (0.90-1.18)	1.04 (0.90-1.20)
**Antiarrhythmics, TSDDs**					
Nonuse	58 720 (99.9)	225 402 (99.9)	1 [Reference]	1 [Reference]	1 [Reference]
1-90	9 (0)	37 (0)	0.88 (0.42-1.84)	0.88 (0.42-1.84)	0.74 (0.33-1.64)
91-365	7 (0)	20 (0)	1.38 (0.58-3.28)	1.35 (0.57-3.22)	1.25 (0.44-3.53)
366-1095	10 (0)	27 (0)	1.22 (0.58-2.56)	1.16 (0.55-2.46)	1.22 (0.56-2.66)
>1095	23 (0)	88 (0)	0.97 (0.61-1.55)	0.99 (0.62-1.58)	0.94 (0.56-1.55)
**Antiepileptics, TSDDs**					
Nonuse	57 358 (97.6)	221 082 (98)	1 [Reference]	1 [Reference]	1 [Reference]
1-90	630 (1.1)	2459 (1.1)	0.98 (0.90-1.07)	0.89 (0.81-0.97)	0.88 (0.80-0.97)
91-365	202 (0.3)	592 (0.3)	1.31 (1.12-1.54)	1.17 (1.00-1.38)	1.14 (0.95-1.36)
366-1095	135 (0.2)	359 (0.2)	1.42 (1.16-1.74)	1.25 (1.02-1.53)	1.13 (0.90-1.41)
>1095	444 (0.8)	1082 (0.5)	1.58 (1.41-1.77)	1.44 (1.28-1.61)	1.39 (1.22-1.57)
**Antimuscarinic Bronchodilators, TSDDs**					
Nonuse	54 891 (93.4)	211 578 (93.8)	1 [Reference]	1 [Reference]	1 [Reference]
1-90	1228 (2.1)	4326 (1.9)	1.10 (1.03-1.17)	1.05 (0.99-1.13)	0.99 (0.92-1.07)
91-365	786 (1.3)	2885 (1.3)	1.05 (0.97-1.14)	1.01 (0.93-1.09)	0.97 (0.89-1.06)
366-1095	742 (1.3)	2719 (1.2)	1.07 (0.99-1.16)	1.02 (0.94-1.11)	0.97 (0.88-1.06)
>1095	1122 (1.9)	4066 (1.8)	1.08 (1.01-1.16)	1.05 (0.98-1.13)	0.97 (0.90-1.05)

Abbreviations: OR, odds ratio; TSDD, total standardized daily dose.

^a ^ Adjusted for other anticholinergic drug types and matching by age, sex, general practice, and calendar time.

^b^ Adjusted for other anticholinergic drug types, body mass index, calculated as weight in kilograms divided by height in meters squared, smoking status, alcohol consumption, Townsend deprivation score, ethnic group, coronary heart disease, atrial fibrillation, heart failure, hypertension, hyperlipidemia, diabetes (type 1 and type 2), stroke, transient ischemic attack, subarachnoid hemorrhage, renal disease, asthma, chronic obstructive pulmonary disease, anxiety, depression, bipolar disorder, schizophrenia, severe head injury, cognitive decline/memory loss, antihypertensive drugs, aspirin, hypnotics, anxiolytic drugs, nonsteroidal antiinflammatory drugs, statins, and matching by age, sex, general practice, and calendar time.

The population-attributable fraction associated with total anticholinergic drug exposure during the 1 to 11 years before diagnosis was 10.3%. For the 3 to 13 years before diagnosis, it was 9.0%, and it was 9.7% for the 5 to 20 years before diagnosis.

### Additional Analyses

There were stronger associations in case patients diagnosed before age 80 years than at 80 years or older, both for total drug exposure and for antidepressants, antipsychotics, and bladder antimuscarinics (eTable 8 in the Supplement). For total cumulative exposure, the AOR for more than 1095 TSDDs was 1.81 (95% CI, 1.71-1.91) in cases diagnosed before age 80 years, whereas it was 1.35 (95% CI, 1.30-1.40) in cases diagnosed at 80 years or older. Associations were similar in men and women (eTable 9 in the Supplement).

Adjusted odds ratios were generally higher for vascular dementia than Alzheimer disease (eTable 10 in the Supplement); for example, in the 1- to 11-year exposure window, the AOR for more than 1095 TSDDs was 1.68 (95% CI, 1.57-1.79) for vascular dementia, and 1.37 (95% CI, 1.30-1.44) for Alzheimer disease.

Results were similar when DDD values were used to calculate cumulative exposure (eTable 12 in the Supplement). Sensitivity analyses using multiply imputed data (eTable 13 in the Supplement) or restricted to anticholinergic drugs included in the study by Gray et al^11^ (eTable 14 in the Supplement) did not change study findings.

## Discussion

This large, nested case-control study found an increased risk of dementia associated with anticholinergic medication use. Associations were strongest for the anticholinergic antidepressants, bladder antimuscarinics, antipsychotics, and antiepileptic drugs. Associations were also stronger in cases diagnosed before the age of 80 years and in cases diagnosed with vascular dementia rather than with Alzheimer disease. There were no significantly increased risks for antihistamines, gastrointestinal antispasmodics, antimuscarinic bronchodilators, antiarrhythmics, or skeletal muscle relaxants, although the numbers of patients prescribed skeletal muscle relaxants and antiarrhythmic drugs were small, giving imprecise estimates.

There was nearly a 50% increased odds of dementia associated with total anticholinergic exposure of more than 1095 TSDDs within a 10-year period, which is equivalent to 3 years’ daily use of a single strong anticholinergic medication at the minimum effective dose recommended for older people. This observational study has shown associations, but is not able to evaluate causality. However, if this association is causal, the population-attributable fractions indicate that around 10% of dementia diagnoses are attributable to anticholinergic drug exposure, which would equate, for example, to around 20 000 of the 209 600 new cases of dementia per year in the United Kingdom.^32^ This proportion is sizeable and is comparable with estimates for other modifiable risk factors for dementia, such as 5% for midlife hypertension, 3% for diabetes, 14% for later-life smoking, and 6.5% for physical inactivity.^1^

The finding of more pronounced associations for vascular dementia than for other types is novel. It raises questions about the mechanisms by which anticholinergic drugs may increase the risk of subsequent dementia. These may include vascular and inflammatory changes,^33,34^ as well as the more obvious mechanism of chronic cholinergic depletion. Perhaps the mechanism underlying the potential effects of anticholinergic drugs is not solely through blocking acetylcholine and causing an excess of Alzheimer disease, so future research should give consideration to possible mechanisms.

We included a large representative sample of people diagnosed with dementia and matched controls. All eligible case patients and controls were included, so there is no selection bias due to nonresponse, and data were recorded prospectively, so results are not susceptible to recall bias. Comprehensive data on prescriptions meant that we could derive a measure of total anticholinergic drug exposure, which accounted for the quantity and dose prescribed.

Our findings are consistent with other studies, including a US cohort study of 3434 participants,^11^ which reported a hazard ratio of 1.54 (95% CI, 1.21-1.96) for the highest exposure category (>1095 TSDDs), similar to our AOR of 1.49 (95% CI, 1.44-1.54). With our larger sample size we could also examine specific types of anticholinergic drugs and account for a broader range of confounders. A study by Richardson et al,^12^ using another United Kingdom primary care database (CPRD), reported findings similar to ours, despite some differences in the drugs included, exposure measures used, exposure windows, and the confounding variables accounted for. For example, we included drugs based on those identified as having strong anticholinergic properties by the American Geriatrics Society 2012 Beers Criteria Update Expert Panel,^14^ whereas Richardson et al^12^ used drugs included in the 2012 update of the Anticholinergic Cognitive Burden scale.^35^ While both CPRD and QResearch are large United Kingdom databases, QResearch is the most nationally representative, while CPRD is more geographically restricted.^36^ Despite these differences, Richardson et al^12^ also found increases in dementia risk for the groups of antidepressant, urological, and antiparkinson drugs considered and no associations for gastrointestinal or antihistamine drugs. The coherence of findings in these 3 studies provides strong evidence for reliability and robustness of the associations across different study designs, countries, and settings. Nevertheless, the possibility of residual confounding remains, and it is impossible to entirely exclude protopathic effects arising from treatment for very early preclinical effects of dementia.

### Limitations

A limitation is that some patients may not have taken their prescribed medication or not taken the dose prescribed, leading to exposure misclassification. This misclassification, if nondifferential, would tend to reduce ORs and might explain the lack of association for antihistamines and the highest exposure category of antivertigo drugs. Our identification of patients with dementia was based on recorded diagnoses or treatment with acetylcholinesterase-inhibiting drugs rather than screening of the entire study population. This means that there will be underascertainment of dementia cases, so some controls may have had undiagnosed dementia, which again would tend to underestimate associations with drug exposure.

The analysis accounted for a wide range of potential confounding variables, but in an observational study, there is potential for residual confounding and indication bias. We endeavored to reduce protopathic bias by excluding prescriptions in the year before diagnosis and in the 3 and 5 years before diagnosis in sensitivity analyses. The increased risks identified for specific drug groups in the main analysis remained in these sensitivity analyses except for the association with antipsychotic drugs, which was not significant when prescriptions in the 5 years before diagnosis were excluded, suggesting that the association may be due to protopathic bias. Some bias due to prescriptions for prodromal symptoms occurring more than 5 years before diagnosis may remain because, while there is an average of 1 to 5 years between onset of symptoms and dementia diagnosis,^37,38^ some early symptoms such as cognitive decline and depression can start to emerge up to 10 years before diagnosis.^39,40^ There is ongoing debate, however, as to whether depression is a risk factor for dementia rather than a prodromal symptom.^1,41^

## Conclusions

The present study adds further evidence of potential risks associated with strong anticholinergic drugs, particularly those that are antidepressants, bladder antimuscarinic drugs, antiparkinson drugs, and epilepsy drugs. Adverse effects should be considered alongside benefits when these drugs are prescribed, and alternative treatments should be considered where possible, such as other types of antidepressant or nonpharmacological treatments for depression, alternative antiparkinsonian drugs, and bladder training or mirabegron for overactive bladders.^42,43^ We found greater increases in risk associated with people diagnosed with dementia before the age of 80, which indicates that anticholinergic drugs should be prescribed with caution in middle-aged and older people.

## Notes

Supplement. **eFigure 1.** Selection of cases and controls for the analysis based on exposures in the 1 to 11 years before index date. **eFigure 2.** Proportions of cases and controls prescribed different types of anticholinergic drug in the 1 to 11 years before diagnosis. **eTable 1.** Anticholinergic drugs included as exposures. **eTable 2.** Recording of dementia type and recorded source of diagnosis in cases. **eTable 3.** Numbers of cases and controls prescribed individual anticholinergic drugs in the 1 to 11 years before the index date. **eTable 4.** Numbers of cases and controls prescribed different types of anticholinergic drugs in the 3 to 13 years before the index date. **eTable 5.** Numbers of cases and controls prescribed different types of anticholinergic drugs in the 5 to 20 years before the index date. **eTable 6.** Adjusted odds ratios for cumulative use of anticholinergic drug types in the 1 to 11, 3 to 13 and 5 to 20 years before the index date. **eTable 7.** Numbers of cases and controls prescribed anticholinergic drug types in the 1 to 11, 3 to 13 and 5 to 20 years before the index date. **eTable 8.** Adjusted odds ratios for cumulative use of anticholinergic drugs in the 1 to 11 years before index date: separate analyses in cases aged less than 80 at diagnosis of dementia and cases aged 80 and over at diagnosis, with their respective matched controls. **eTable 9.** Adjusted odds ratios for cumulative use of anticholinergic drugs in the 1 to 11 years before the index date: separate analyses in men and women, with their respective matched controls. **eTable 10.** Adjusted odds ratios for cumulative use of anticholinergic drugs in the 1 to 11 years before index date: separate analyses in cases with Alzheimer’s, cases with vascular dementia, and cases with other types or unspecified type of dementia with their respective matched controls. **eTable 11.** Adjusted odds ratios for total cumulative use in DDDs of anticholinergic drugs in 1 to 11, 3 to 13 and 5 to 20 years before the index date. **eTable 12.** Adjusted odds ratios for cumulative use of anticholinergic drugs in 1 to11 years before index date: multiple imputation results. **eTable 13.** Adjusted odds ratios for cumulative use of anticholinergic drugs in the 1 to 11 years before index date: restricted to the anticholinergic drugs included in Gray study.
