# Supplementary material for: Anticholinergic Drug Exposure and the Risk of Dementia: A Nested Case-Control Study
Source: JAMA Intern Med. 2019 Jun 24;179(8):1084–93. doi: 10.1001/jamainternmed.2019.0677 (PMC6593623; doi:10.1001/jamainternmed.2019.0677)
Supplement: Supplement. — eFigure 1. Selection of cases and controls for the analysis based on exposures in the 1 to 11 years before index date. eFigure 2. Proportions of cases and controls prescribed different types of anticholinergic drug in the 1 to 11 years before diagnosis. eTable 1. Anticholinergic drugs included as exposures. eTable 2. Recording of dementia type and recorded source of diagnosis in cases. eTable 3. Numbers of cases and controls prescribed individual anticholinergic drugs in the 1 to 11 years before the index date. eTable 4. Numbers of cases and controls prescribed different types of anticholinergic drugs in the 3 to 13 years before the index date. eTable 5. Numbers of cases and controls prescribed different types of anticholinergic drugs in the 5 to 20 years before the index date. eTable 6. Adjusted odds ratios for cumulative use of anticholinergic drug types in the 1 to 11, 3 to 13 and 5 to 20 years before the index date. eTable 7. Numbers of cases and controls prescribed anticholinergic drug types in the 1 to 11, 3 to 13 and 5 to 20 years before the index date. eTable 8. Adjusted odds ratios for cumulative use of anticholinergic drugs in the 1 to 11 years before index date: separate analyses in cases aged less than 80 at diagnosis of dementia and cases aged 80 and over at diagnosis, with their respective matched controls. eTable 9. Adjusted odds ratios for cumulative use of anticholinergic drugs in the 1 to 11 years before the index date: separate analyses in men and women, with their respective matched controls. eTable 10. Adjusted odds ratios for cumulative use of anticholinergic drugs in the 1 to 11 years before index date: separate analyses in cases with Alzheimer’s, cases with vascular dementia, and cases with other types or unspecified type of dementia with their respective matched controls. eTable 11. Adjusted odds ratios for total cumulative use in DDDs of anticholinergic drugs in 1 to 11, 3 to 13 and 5 to 20 years before the index date. eTable 12. Adjusted o [file jamainternmed-179-1084-s001.pdf]

Supplementary Online Content

Coupland CAC, Hill T, Dening T, Morriss R, Moore M, Hippisley-Cox J. Anticholinergenic drug exposure and the risk of dementia: a nested case-control study. *JAMA Intern Med*. Published online June 24, 2019. doi:10.1001/ jamainternalmed.2019.0677.

**eFigure 1**	Selection of cases and controls for the analysis based on exposures in the 1 to 11 years before index date
**eFigure 2**	Proportions of cases and controls prescribed different types of anticholinergic drug in the 1 to 11 years before diagnosis
**eTable 1**	Anticholinergic drugs included as exposures
**eTable 2**	Recording of dementia type and recorded source of diagnosis in cases
**eTable 3**	Numbers of cases and controls prescribed individual anticholinergic drugs in the 1 to 11 years before the index date
**eTable 4**	Numbers of cases and controls prescribed different types of anticholinergic drugs in the 3 to 13 years before the index date
**eTable 5**	Numbers of cases and controls prescribed different types of anticholinergic drugs in the 5 to 20 years before the index date
**eTable 6**	Adjusted odds ratios for cumulative use of anticholinergic drug types in the 1 to 11, 3 to 13 and 5 to 20 years before the index date
**eTable 7**	Numbers of cases and controls prescribed anticholinergic drug types in the 1 to 11, 3 to 13 and 5 to 20 years before the index date
**eTable 8**	Adjusted odds ratios for cumulative use of anticholinergic drugs in the 1 to 11 years before index date: separate analyses in cases aged less than 80 at diagnosis of dementia and cases aged 80 and over at diagnosis, with their respective matched controls
**eTable 9**	Adjusted odds ratios for cumulative use of anticholinergic drugs in the 1 to 11 years before the index date: separate analyses in men and women, with their respective matched controls
**eTable 10**	Adjusted odds ratios for cumulative use of anticholinergic drugs in the 1 to 11 years before index date: separate analyses in cases with Alzheimer's, cases with vascular dementia, and cases with other types or unspecified type of dementia with their respective matched controls
**eTable 11**	Adjusted odds ratios for total cumulative use in DDDs of anticholinergic drugs in 1 to 11, 3 to 13 and 5 to 20 years before the index date
**eTable 12**	Adjusted odds ratios for cumulative use of anticholinergic drugs in 1 to 11 years before index date: multiple imputation results

© 2019 Coupland et al. JAMA Internal Medicine

eTable 13 Adjusted odds ratios for cumulative use of anticholinergic drugs in the 1
to 11 years before index date: restricted to the anticholinergic drugs
included in Gray study

This supplementary material has been provided by the authors to give readers additional information about their work.

© 2019 Coupland et al. JAMA Internal Medicine

eFigure 1 Selection of cases and controls for the analysis based on exposures in the 1 to 11 years before index date

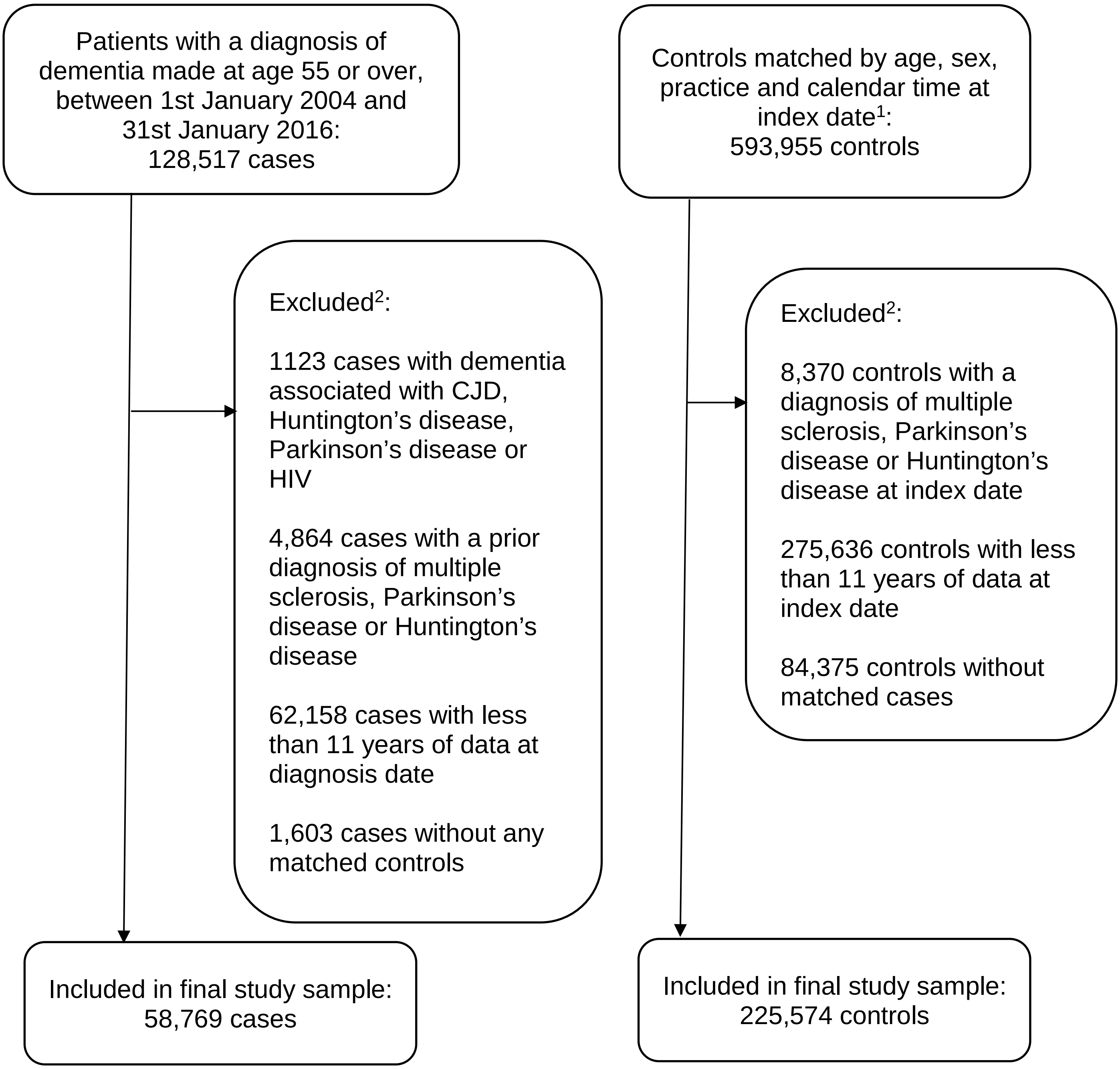

[1] Index date in controls is date of diagnosis of dementia in their matched cases
[2] Exclusion criteria were applied in the order shown

© 2019 Coupland et al. JAMA Internal Medicine

eFigure 2 Proportions of cases and controls prescribed different types of anticholinergic drug in the 1 to 11 years before diagnosis

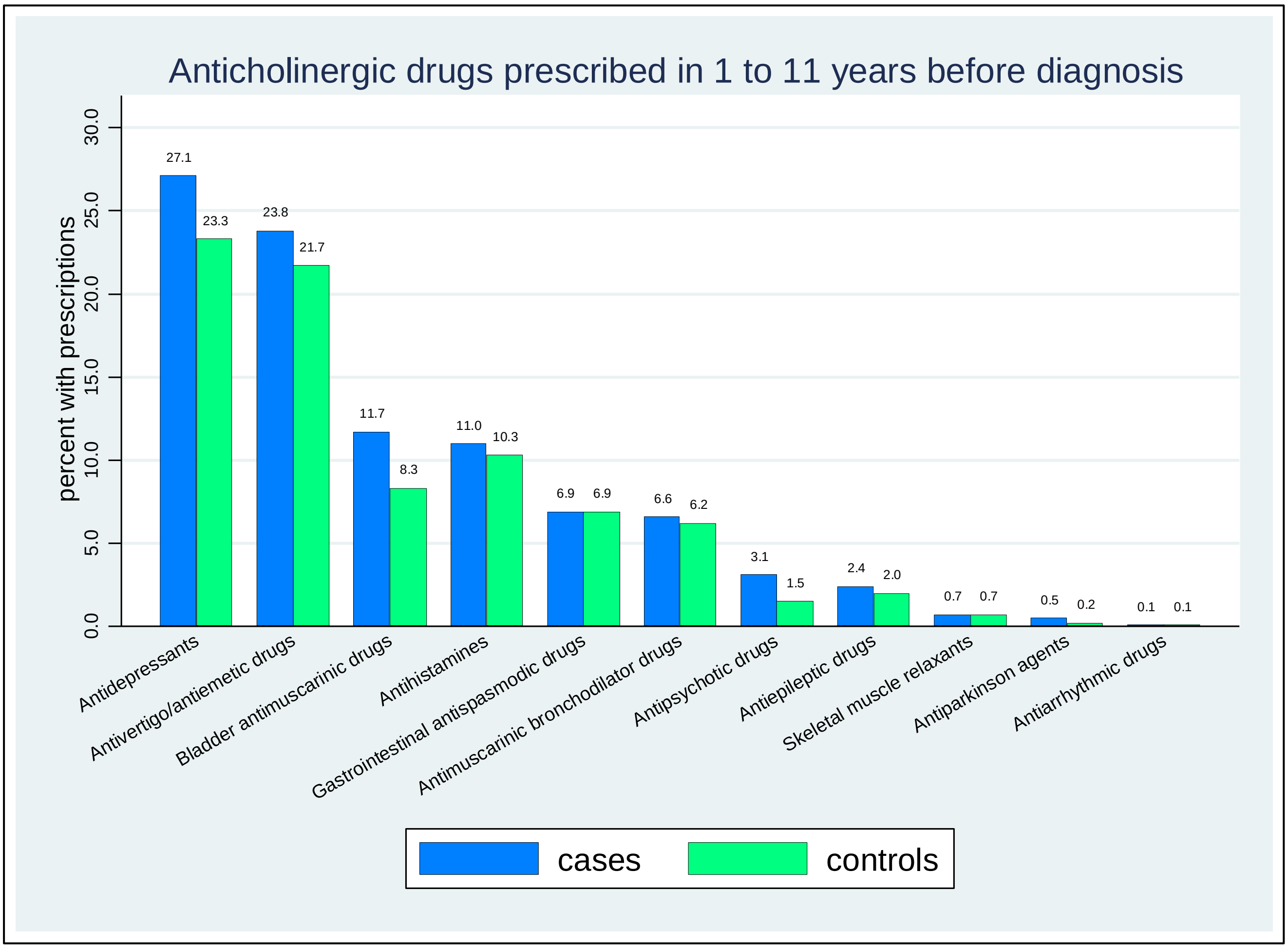

© 2019 Coupland et al. JAMA Internal Medicine

eTable 1 Anticholinergic drugs included as exposures

Drug name and type	Basis for inclusion	Minimum effective daily dose (MEDD) in older people[a]	Defined daily dose (DDD)[b]
Antiarrhythmic drugs			
Disopyramide	Gray Criteria[c]	400 mg	400 mg
Antihistamines			
Azatadine	Gray Criteria[c]	2 mg	2 mg
Brompheniramine	Gray Criteria[c]	12 mg	24 mg
Chlorpheniramine (Chlorphenamine)	Gray Criteria[c]	4 mg	12 mg
Clemastine	Gray Criteria[c]	2 mg	2 mg
Cyproheptadine	Gray Criteria[c]	4 mg	12 mg
Diphenhydramine	Gray Criteria[c]	50 mg	200 mg
Hydroxyzine	Gray Criteria[c]	75 mg	75 mg
Trimeprazine (Alimemazine)	Gray Criteria[c]	10 mg	30 mg
Antidepressants			
Amitriptyline	Gray Criteria[c]	10 mg	75 mg
Clomipramine	Gray Criteria[c]	25 mg	100 mg
Dosulepin	Review of BNF/ summary of product characteristics	75 mg[f]	150 mg
Doxepin	Gray Criteria[c]	10 mg	100 mg
Imipramine	Gray Criteria[c]	10 mg	100 mg
Lofepramine	Review of BNF/ summary of product characteristics	70 mg[f]	105 mg
Nortiptyline	Gray Criteria[c]	10 mg	75 mg
Paroxetine	Gray Criteria[c]	10 mg	20 mg
Trimipramine	Gray Criteria[c]	50 mg	150 mg
Antiepileptics			
Carbamazepine	Included in ACB scale[e]	200 mg[f]	1000 mg
Oxcarbazepine	Included in ACB scale[e]	600 mg[f]	1000 mg
Antivertigo/antiemetic			
Cyclizine	Gray Criteria[c]	50 mg	100 mg
Dimenhydrinate	Gray Criteria[c]	200 mg	200 mg[h]
Prochlorperazine	Gray Criteria[c]	15 mg	100 mg
Promethazine	Gray Criteria[c]	50 mg	25 mg
Promazine	Gray Criteria[c]	25 mg[f]	300 mg (tablet)<br>100 mg (parenteral)
Antiparkinson agents			
Benztropine (Benzatropine)	Gray Criteria[c]	0.5 mg	2 mg

© 2019 Coupland et al. JAMA Internal Medicine

Orphenadrine	Gray Criteria[c]	200 mg	200 mg (chloride)<br>120 mg (citrate)
Procyclidine	Gray Criteria[c]	7.5 mg	25 mg
Trihexyphenidyl	Gray Criteria[c]	6 mg	10 mg
Antipsychotics			
Chlorpromazine	Gray Criteria[c]	10 mg	300 mg (oral)<br>100 mg (parenteral)
Clozapine	Gray Criteria[c]	20 mg	300 mg
Methotrimeprazine (levomepromazine)	Classified as high potency anticholinergic in Duran review[d] and included in ACB scale[e]	25 mg[f]	300 mg
Olanzapine	Gray Criteria[c]	2.5 mg	10 mg
Pericyazine	Review of BNF/summary of product characteristics	10 mg[f]	50 mg
Perphenazine	Classified as high potency anticholinergic in Duran review[d] and included in ACB scale[e]	4 mg[f]	30 mg (oral)
Pimozide	Gray Criteria[c]	1 mg	4 mg
Quetiapine	Included in ACB scale[e]	25 mg[f]	400 mg
Thioridazine	Gray Criteria[c]	10 mg	300 mg
Trifluoperazine	Gray Criteria[c]	0.5 mg	20 mg
Bladder antimuscarinics			
Darifenacin	Gray Criteria[c]	7.5 mg	7.5 mg
Fesoterodine	Gray Criteria[c]	4 mg[f]	4 mg
Flavoxate	Gray Criteria[c]	300 mg	800 mg
Oxybutynin	Gray Criteria[c]	5 mg (oral)<br>3.9 mg (patch)	15 mg (oral)<br>3.9 mg (transdermal)
Propiverine	Included in ACB scale[e]	15 mg[f]	30 mg
Solifenacin	Gray Criteria[c]	5 mg	5 mg
Tolterodine	Gray Criteria[c]	2 mg	4 mg
Trospium	Gray Criteria[c]	20 mg	40 mg
Skeletal muscle relaxants			
Methocarbamol	Included in ACB scale[e]	2000 mg[f]	3000 mg
Tizanidine	Classified as high potency anticholinergic in Duran review[d]	2 mg[f]	12 mg
Gastrointestinal antispasmodics			

© 2019 Coupland et al. JAMA Internal Medicine

Alverine	Gray Criteria[c]	60 mg[f]	240 mg[h]
Atropine products	Gray Criteria[c]	0.0582 mg	1.5 mg
Dicyclomine (Dicycloverine)	Gray Criteria[c]	40 mg	80 mg
Propantheline	Gray Criteria[c]	22.5 mg	60 mg
Scopolamine (hyoscine)	Gray Criteria[c]	0.0195 mg (oral)<br>0.33 mg (patch)<br>30 mg[f] (butylscopolamine form)	0.9 mg (hydrobromide form)<br>60 mg (butylbromide form)
Antimuscarinic bronchodilator drugs			
Glycopyrrolate (Glycopyrronium)	Gray Criteria[c]	0.6 mg (oral)<br>0.044 mg[g] (inhaled powder)	3 mg (oral)<br>0.044 mg (inhaled powder)
Ipratropium	Classified as high potency anticholinergic in Duran review[d]	0.24 mg[g] (nasal)<br>0.12 mg[g] (inhalation-aerosol)<br>0.3 mg[g] (inhalation - solution)	0.24 mg (nasal)<br>0.12 mg (inhalation-aerosol)<br>0.3 mg (inhalation - solution)

BNF = British National Formulary

[a] Minimum effective daily dose (MEDD) recommended for use in older adults obtained from the Geriatric Dosage Handbook (Semla TP, Beizer JL, MD. H. Geriatric Dosage Handbook. 15th edition. Hudson, Ohio : Lexi-Comp, 2010)

[b] Defined daily dose (DDD) values assigned by the World Health Organisation's (WHO) Collaborating Centre for Drug Statistics Methodology (https://www.whocc.no/atc_ddd_index/)

[c] These drugs were included in the Gray study (Gray SL, Anderson ML, Dublin S, et al. Cumulative use of strong anticholinergics and incident dementia: A prospective cohort study. JAMA Internal Medicine 2015;175:401-07). They include drugs identified as having strong anticholinergic properties by the American Geriatrics Society 2012 Beers Criteria Update Expert Panel and some further drugs identified as strong anticholinergic drugs. Restricted to drugs which have been prescribed in England (based on 1998/2014 Prescription Cost Analysis data).

[d] Additional drug included based on assessment in Duran review (Durán C, Azermai M, Vander Stichele R. Systematic review of anticholinergic risk scales in older adults. Eur J Clin Pharmacol 2013;69:1485-96.)

[e] Additional drug included based on a score of 2 or 3 in the Anti-Cholinergic Burden (ACB) Scale (Boustani M, Campbell N, Munger S, et al. Impact of anticholinergics on the aging brain: a review and practical application. Aging Health 2008;4:311-20.)

[f] Value is lowest recommended dose in older people obtained from BNF

[g] DDD value used as a MEDD value was not available

[h] Value is recommended dose obtained from BNF as a DDD value was not available

© 2019 Coupland et al. JAMA Internal Medicine

eTable 2 Recording of dementia type and recorded source of diagnosis in cases

	Cases (N=58,769)
	N (%)
**Dementia type**	
Alzheimer's (including mixed)	22,034 (60.1)[a]
Vascular dementia	13,313 (36.3)[a]
Other types	1319 (3.6)[a]
Dementia type unspecified/not known	19,024
Dementia drug/death code only	3079
**Recording of dementia diagnosis**	
Diagnosis code in GP record only	41,535 (70.7)
Dementia drug prescription only	1362 (2.3)
Death record code only	1640 (2.8)
GP diagnosis code and dementia drug prescription	11,202 (19.1)
GP diagnosis code and death record code	2196 (3.7)
Dementia drug prescription and death record code	77 (0.1)
GP diagnosis code, dementia drug prescription and death record code	757 (1.3)

[a] % is out of 36,666 cases with dementia type recorded

© 2019 Coupland et al. JAMA Internal Medicine

eTable 3 Numbers of cases and controls prescribed individual anticholinergic drugs in the 1 to 11 years before the index date

	Cases (N=58,769)				Controls (N=225,574)			
**Drug name and type**	**Number with prescriptions (%)**	**Total number of prescriptions (%)**	**Median number of prescriptions[a] (IQR)**	**Median cumulative dose[ab] (IQR)**	**Number with prescriptions (%)**	**Total number of prescriptions (%)**	**Median number of prescriptions[a] (IQR)**	**Median cumulative dose[ab] (IQR)**
**Antihistamines**								
Azatadine	2 (0)	20 (0)	10 (1, 19)	805 (14, 1596)	3 (0)	3 (0)	1 (1, 1)	28 (10, 28)
Brompheniramine	331 (0.6)	871 (0.1)	1 (1, 2)	13 (13, 28)	1312 (0.6)	2973 (0.1)	1 (1, 2)	13 (13, 28)
Chlorpheniramine	4801 (8.2)	20175 (2.1)	1 (1, 2)	30 (28, 84)	17249 (7.7)	66995 (2.7)	1 (1, 2)	30 (28, 84)
Clemastine	42 (0.1)	238 (0)	1 (1, 2)	23 (15, 60)	151 (0.1)	1202 (0)	1 (1, 3)	30 (15, 90)
Cyproheptadine	37 (0.1)	185 (0)	1 (1, 2)	60 (30, 126)	81 (0)	634 (0)	1 (1, 5)	60 (30, 330)
Diphenhydramine	136 (0.2)	879 (0.1)	1 (1, 3)	16 (10, 60)	386 (0.2)	2212 (0.1)	1 (1, 2)	16 (10, 32)
Hydroxyzine	1734 (3)	10418 (1.1)	1 (1, 3)	12 (9, 37)	6071 (2.7)	36621 (1.5)	1 (1, 3)	13 (9, 37)
Trimeprazine	177 (0.3)	1365 (0.1)	1 (1, 3)	56 (28, 112)	643 (0.3)	6631 (0.3)	1 (1, 4)	56 (28, 164)
**Antidepressants**								
Amitriptyline	11533 (19.6)	225964 (23.7)	3 (1, 19)	168 (56, 1190)	40423 (17.9)	626136 (25.0)	2 (1, 12)	140 (56, 784)
Clomipramine	294 (0.5)	10448 (1.1)	8 (1, 64)	522 (44, 4116)	726 (0.3)	25085 (1.0)	9 (1, 60)	417 (34, 3136)
Dosulepin	3183 (5.4)	89877 (9.4)	6 (1, 39)	140 (28, 1020)	9294 (4.1)	240513 (9.6)	5 (1, 36)	123 (28, 931)
Doxepin	280 (0.5)	3861 (0.4)	2 (1, 13)	251 (70, 1582)	922 (0.4)	14396 (0.6)	2 (1, 14)	294 (140, 1610)
Imipramine	472 (0.8)	9493 (1.0)	3 (1, 25)	251 (70, 2800)	1329 (0.6)	28661 (1.1)	3 (1, 25)	232 (70, 3000)
Lofepramine	1059 (1.8)	15065 (1.6)	2 (1, 10)	112 (56, 560)	2655 (1.2)	34489 (1.4)	2 (1, 8)	84 (56, 448)
Nortriptyline	576 (1)	8483 (0.9)	3 (1, 12)	158 (56, 684)	1935 (0.9)	24809 (1.0)	2 (1, 11)	120 (56, 668)
Paroxetine	1804 (3.1)	55675 (5.8)	8 (2, 41)	600 (120, 3300)	4616 (2.1)	123554 (4.9)	6 (1, 38)	480 (60, 2970)
Trimipramine	195 (0.3)	8623 (0.9)	18 (2, 72)	504 (56, 2644)	567 (0.3)	23641 (0.9)	22 (2, 65)	546 (29, 2128)

© 2019 Coupland et al. JAMA Internal Medicine

	Cases (N=58,769)				Controls (N=225,574)			
**Drug name and type**	**Number with prescriptions (%)**	**Total number of prescriptions (%)**	**Median number of prescriptions[a] (IQR)**	**Median cumulative dose[ab] (IQR)**	**Number with prescription s (%)**	**Total number of prescriptions (%)**	**Median number of prescriptions[a] (IQR)**	**Median cumulative dose[ab] (IQR)**
**Antivertigo/antiemetic drugs**								
Cyclizine	1493 (2.5)	7524 (0.8)	1 (1, 2)	50 (28, 100)	4988 (2.2)	19092 (0.8)	1 (1, 2)	42 (21, 100)
Dimenhydrinate	26 (0)	135 (0)	2 (1, 2)	20 (15, 40)	87 (0)	607 (0)	1 (1, 7)	20 (20, 138)
Prochlorperazine	12784 (21.8)	65895 (6.9)	2 (1, 3)	19 (9, 43)	45099 (20.0)	211860 (8.5)	1 (1, 3)	19 (9, 39)
Promazine	135 (0.2)	1340 (0.1)	2 (1, 10)	140 (42, 540)	291 (0.1)	4036 (0.2)	2 (1, 10)	90 (30, 510)
Promethazine	587 (1.0)	4779 (0.5)	1 (1, 3)	21 (11, 56)	1832 (0.8)	13619 (0.5)	1 (1, 3)	14 (11, 40)
**Antiparkinson drugs**								
Benzatropine	7 (0)	212 (0)	26 (1, 64)	1384 (120, 16128)	8 (0)	61 (0)	2 (1, 15)	198 (120, 3720)
Orphenadrine	23 (0)	1068 (0.1)	26 (3, 88)	448 (30, 1950)	72 (0)	3086 (0.1)	26 (2, 66)	424 (42, 1273)
Procyclidine	232 (0.4)	12947 (1.4)	29 (3, 91)	837 (112, 3395)	366 (0.2)	17788 (0.7)	23 (2, 73)	589 (67, 2429)
Trihexyphenidyl	58 (0.1)	2271 (0.2)	10 (1, 58)	303 (33, 1745)	109 (0.1)	4477 (0.2)	4 (1, 60)	112 (28, 1560)
**Antipsychotic drugs**								
Chlorpromazine	316 (0.5)	9844 (1.0)	4 (1, 37)	560 (75, 5483)	628 (0.3)	15035 (0.6)	2 (1, 23)	147 (70, 2854)
Clozapine	2 (0)	4 (0)	2 (2, 2)	250 (75, 425)	4 (0)	25 (0)	3 (1, 12)	420 (143, 4445)
Methotrimeprazine	11 (0)	29 (0)	1 (1, 5)	14 (10, 114)	59 (0)	235 (0)	1 (1, 5)	28 (10, 84)
Olanzapine	483 (0.8)	22938 (2.4)	18 (4, 66)	952 (140, 3080)	726 (0.3)	31710 (1.3)	22 (4, 61)	896 (168, 3164)
Pericyazine	17 (0)	460 (0)	2 (1, 44)	28 (21, 287)	21 (0)	522 (0)	4 (2, 34)	60 (15, 427)
Perphenazine	142 (0.2)	4211 (0.4)	16 (2, 53)	392 (45, 1728)	433 (0.2)	15610 (0.6)	21 (3, 60)	585 (60, 1880)
Pimozide	7 (0)	235 (0)	2 (1, 88)	480 (120, 11516)	17 (0)	747 (0)	20 (1, 54)	1120 (112, 3996)
Quetiapine	467 (0.8)	14585 (1.5)	11 (3, 37)	517 (114, 1800)	447 (0.2)	13092 (0.5)	10 (2, 36)	504 (112, 2160)
Thioridazine	325 (0.6)	4550 (0.5)	4 (1, 15)	336 (70, 1800)	688 (0.3)	7838 (0.3)	3 (1, 11)	210 (60, 1080)
Trifluoperazine	419 (0.7)	13039 (1.4)	6 (2, 44)	960 (172, 8800)	920 (0.4)	24366 (1.0)	5 (1, 39)	602 (112, 6024)

© 2019 Coupland et al. JAMA Internal Medicine

	Cases (N=58,769)				Controls (N=225,574)			
Drug name and type	Number with prescriptions (%)	Total number of prescriptions (%)	Median number of prescriptions[a] (IQR)	Median cumulative dose[ab] (IQR)	Number with prescriptions (%)	Total number of prescriptions (%)	Median number of prescriptions[a] (IQR)	Median cumulative dose[ab] (IQR)
**Bladder antimuscarinic drugs**								
Darifenacin	25 (0)	204 (0)	4 (2, 8)	112 (56, 280)	87 (0)	652 (0)	3 (1, 7)	112 (42, 336)
Fesoterodine	145 (0.3)	1434 (0.2)	3 (1, 11)	112 (28, 420)	333 (0.2)	2867 (0.1)	3 (1, 9)	140 (28, 392)
Flavoxate	239 (0.4)	2021 (0.2)	1 (1, 4)	60 (40, 240)	679 (0.3)	5822 (0.2)	1 (1, 4)	60 (53, 210)
Oxybutynin	3632 (6.2)	57685 (6.1)	4 (1, 16)	120 (30, 601)	9985 (4.4)	132527 (5.3)	2 (1, 12)	84 (30, 474)
Propiverine	173 (0.3)	3080 (0.3)	3 (1, 18)	224 (56, 1239)	441 (0.2)	6166 (0.2)	2 (1, 13)	168 (56, 868)
Solifenacin	1954 (3.3)	29954 (3.1)	5 (1, 18)	210 (60, 720)	4859 (2.2)	56883 (2.3)	4 (1, 14)	148 (30, 600)
Tolterodine	3222 (5.5)	68053 (7.1)	5 (1, 25)	308 (56, 1400)	8691 (3.9)	141899 (5.7)	3 (1, 17)	196 (56, 1064)
Trospium	587 (1.0)	7633 (0.8)	3 (1, 16)	240 (60, 900)	1483 (0.7)	15861 (0.6)	2 (1, 9)	120 (60, 600)
**Skeletal muscle relaxants**								
Methocarbamol	418 (0.7)	1105 (0.1)	1 (1, 2)	23 (16, 42)	1521 (0.7)	4141 (0.2)	1 (1, 2)	23 (16, 42)
Tizanidine	11 (0)	256 (0)	7 (1, 47)	960 (84, 4200)	53 (0)	1061 (0)	2 (1, 11)	148 (60, 1120)
**Gastrointestinal antispasmodic drugs**								
Alverine	780 (1.3)	7671 (0.8)	2 (1, 6)	128 (100, 600)	3077 (1.4)	28730 (1.1)	2 (1, 5)	144 (100, 500)
Atropine	888 (1.5)	4852 (0.5)	1 (1, 2)	21 (13, 52)	2938 (1.3)	15516 (0.6)	1 (1, 2)	21 (13, 47)
Dicyclomine	979 (1.7)	6749 (0.7)	1 (1, 3)	15 (6, 44)	3774 (1.7)	25292 (1.0)	1 (1, 3)	19 (7, 48)
Propantheline	92 (0.2)	1257 (0.1)	1 (1, 11)	75 (40, 476)	286 (0.1)	2574 (0.1)	1 (1, 6)	75 (37, 300)
Scopolamine	1875 (3.2)	8791 (0.9)	1 (1, 2)	19 (14, 56)	7506 (3.3)	29156 (1.2)	1 (1, 2)	19 (13, 37)
**Antiarrhythmic drugs**								
Disopyramide	49 (0.1)	2569 (0.3)	31 (5, 88)	882 (175, 2345)	172 (0.1)	8142 (0.3)	37 (5, 77)	1148 (150, 2436)

© 2019 Coupland et al. JAMA Internal Medicine

	Cases (N=58,769)				Controls (N=225,574)			
**Drug name and type**	**Number with prescriptions (%)**	**Total number of prescriptions (%)**	**Median number of prescriptions[a] (IQR)**	**Median cumulative dose[ab] (IQR)**	**Number with prescription s (%)**	**Total number of prescriptions (%)**	**Median number of prescriptions[a] (IQR)**	**Median cumulative dose[ab] (IQR)**
**Antiepileptic drugs**								
Carbamazepine	1405 (2.4)	40929 (4.3)	4 (1, 39)	148 (42, 2212)	4479 (2.0)	96884 (3.9)	2 (1, 20)	80 (30, 959)
Oxcarbazepine	10 (0)	431 (0)	17 (3, 99)	536 (38, 4032)	27 (0)	296 (0)	2 (1, 10)	30 (21, 273)
**Antimuscarinic bronchodilator drugs**								
Glycopyrrolate	5 (0)	5 (0)	1 (1, 1)	35 (30, 38)	47 (0)	288 (0)	4 (1, 9)	167 (38, 360)
Ipratropium	3877 (6.6)	79878 (8.4)	8 (2, 29)	300 (60, 1330)	13960 (6.2)	287672 (11.5)	8 (2, 29)	331 (67, 1333)

[a] In patients with one or more prescriptions for drug

[b] Cumulative dose calculated using total standardized daily doses (TSDDs) in exposure window.

© 2019 Coupland et al. JAMA Internal Medicine

eTable 4 Numbers of cases and controls prescribed different types of anticholinergic drugs in the 3 to 13 years before the index date

	Cases (N=45,621)				Controls (N=169,020)			
**Anticholinergic drug group**	**Number with prescriptions (%)**	**Total prescriptions (%)**	**Median number of prescriptions[a] (IQR)**	**Median cumulative dose[ab] (IQR)**	**Number with prescriptions (%)**	**Total prescriptions (%)**	**Median number of prescriptions[a] (IQR)**	**Median cumulative dose[ab] (IQR)**
Any anticholinergic drugs	25076 (55.0)	626166 (100)	5 (2, 28)	185 (40, 1350)	84344 (49.9)	1657509 (100)	4 (1, 19)	126 (30, 865)
Antihistamines	4943 (10.8)	23546 (3.8)	1 (1, 2)	30 (25, 81)	17078 (10.1)	75272 (4.5)	1 (1, 2)	30 (22, 77)
Antidepressants	12225 (26.8)	293940 (46.9)	5 (1, 31)	280 (65, 1764)	38539 (22.8)	773224 (46.6)	4 (1, 23)	196 (56, 1268)
Antivertigo/antiemetic drugs	10536 (23.1)	54361 (8.7)	2 (1, 3)	20 (9, 56)	35918 (21.3)	170113 (10.3)	1 (1, 3)	18.67 (9, 48)
Antiparkinson agents	222 (0.5)	10902 (1.7)	26 (3, 78)	690 (75, 3127)	415 (0.2)	18545 (1.1)	16 (2, 69)	504 (43, 2244)
Antipsychotic drugs	1223 (2.7)	42542 (6.8)	9 (2, 50)	708 (112, 4480)	2721 (1.6)	74266 (4.5)	6 (1, 38)	420 (80, 2590)
Bladder antimuscarinic drugs	4578 (10.0)	92406 (14.8)	6 (2, 25)	252 (56, 1241)	12352 (7.3)	204732 (12.4)	4 (1, 19)	168 (56, 952)
Skeletal muscle relaxants	355 (0.8)	969 (0.2)	1 (1, 2)	30 (16, 42)	1224 (0.7)	3630 (0.2)	1 (1, 2)	22.5 (16, 42)
Gastrointestinal antispasmodic drugs	3205 (7.0)	21579 (3.4)	1 (1, 4)	30 (13, 120)	11365 (6.7)	70133 (4.2)	1 (1, 3)	28 (13, 112)
Antiarrhythmic drugs	41 (0.1)	2007 (0.3)	36 (11, 74)	1320 (330, 2044)	143 (0.1)	6413 (0.4)	34 (4, 73)	1020 (105, 2478)
Antiepileptic drugs	1140 (2.5)	26150 (4.2)	3 (1, 29)	112 (42, 1652)	3408 (2)	64233 (3.9)	2 (1, 15)	84 (30, 777)
Antimuscarinic bronchodilator drugs	2922 (6.4)	57764 (9.2)	8 (2, 27)	300 (63, 1262)	9999 (5.9)	196948 (11.9)	7 (2, 28)	300 (60, 1267)

[a] In patients with one or more prescriptions for drug

[b] Cumulative dose calculated using total standardised daily doses (TSDDs) in exposure window.

© 2019 Coupland et al. JAMA Internal Medicine

eTable 5 Numbers of cases and controls prescribed different types of anticholinergic drugs in the 5 to 20 years before the index date

	**Cases (N=8,283)**				**Controls (N=27,200)**			
**Anticholinergic drug group**	**Number with prescriptions (%)**	**Total prescriptions (%)**	**Median number of prescriptions[a] (IQR)**	**Median cumulative dose[ab] (IQR)**	**Number with prescriptions (%)**	**Total prescriptions (%)**	**Median number of prescriptions[a] (IQR)**	**Median cumulative dose[ab] (IQR)**
Any anticholinergic drugs	4948 (59.7)	129441 (100)	5 (2, 26)	189 (40, 1269)	14919 (54.8)	318689 (100)	4 (1, 18)	130 (30, 822)
Antihistamines	1200 (14.5)	5157 (4.0)	1 (1, 2)	30 (27, 84)	3445 (12.7)	14727 (4.6)	1 (1, 2)	30 (24, 75)
Antidepressants	2512 (30.3)	64561 (49.9)	5 (2, 29)	300 (70, 1810)	6988 (25.7)	154150 (48.4)	4 (1, 22)	225 (70, 1344)
Antivertigo/antiemetic drugs	2267 (27.4)	11832 (9.1)	2 (1, 4)	19 (9, 49)	6863 (25.2)	33528 (10.5)	2 (1, 3)	19 (9, 47)
Antiparkinson drugs	48 (0.6)	1723 (1.3)	9 (2, 58)	261 (58, 2053)	89 (0.3)	4538 (1.4)	19 (2, 76)	536 (67, 2769)
Antipsychotic drugs	278 (3.4)	9053 (7.0)	8 (1, 40)	577 (80, 3422)	684 (2.5)	19014 (6)	3 (1, 25)	240 (60, 2415)
Bladder antimuscarinic drugs	831 (10)	14715 (11.4)	5 (2, 20)	240 (57, 1080)	2022 (7.4)	32973 (10.3)	4 (1, 17)	164 (56, 840)
Skeletal muscle relaxants	97 (1.2)	201 (0.2)	1 (1, 2)	30 (19, 42)	251 (0.9)	565 (0.2)	1 (1, 2)	23 (19, 38)
Gastrointestinal antispasmodic drugs	787 (9.5)	4868 (3.8)	1 (1, 3)	28 (13, 100)	2300 (8.5)	12339 (3.9)	1 (1, 3)	26 (13, 100)
Antiarrhythmic drugs	11 (0.1)	598 (0.5)	36 (1, 98)	1152 (21, 4564)	25 (0.1)	1412 (0.4)	53 (2, 81)	2029 (45, 3645)
Antiepileptic drugs	228 (2.8)	5291 (4.1)	2 (1, 24)	84 (33, 1796)	684 (2.5)	11202 (3.5)	2 (1, 8)	69 (30, 425)
Antimuscarinic bronchodilator drugs	538 (6.5)	11442 (8.8)	6 (1, 27)	221 (60, 1157)	1670 (6.1)	34241 (10.7)	1 (1, 2)	30 (24, 75)

[a] In patients with one or more prescriptions for drug

[b] Cumulative dose calculated using total standardised daily doses (TSDDs) in exposure window.

© 2019 Coupland et al. JAMA Internal Medicine

eTable 6 Adjusted odds ratios for cumulative use of anticholinergic drug types in the 1 to 11, 3 to 13 and 5 to 20 years before the index date

Anticholinergic drug type	1 to 11 years before index date		3 to 13 years before index date		5 to 20 years before index date	
	Cases=58,769 Controls=225,574		Cases=45,621 Controls=169,020		Cases=8,283 Controls=27,200	
	OR[a]	95% CI	OR[a]	95% CI	OR[a]	95% CI
**Antihistamines**						
Non-use	1.00		1.00		1.00	
1-90 TSDDs	1.03	(0.99 to 1.07)	1.04	(0.99 to 1.08)	1.09[b]	(1.01 to 1.18)
91-365 TSDDs	1.03	(0.95 to 1.12)	0.98	(0.89 to 1.07)		
366-1095 TSDDs	1.02	(0.88 to 1.18)	1.03	(0.87 to 1.22)		
>1095 TSDDs	1.14	(0.98 to 1.34)	1.18	(0.97 to 1.43)		
**Antidepressants**						
Non-use	1.00		1.00		1.00	
1-90 TSDDs	1.02	(0.98 to 1.06)	1.06	(1.01 to 1.10)	1.11	(1.01 to 1.23)
91-365 TSDDs	1.12	(1.07 to 1.17)	1.13	(1.08 to 1.19)	1.14	(1.02 to 1.27)
366-1095 TSDDs	1.25	(1.18 to 1.32)	1.21	(1.13 to 1.29)	1.20	(1.05 to 1.37)
>1095 TSDDs	1.29	(1.24 to 1.34)	1.31	(1.25 to 1.38)	1.33	(1.20 to 1.48)
**Antivertigo/antiemetic drugs**						
Non-use	1.00		1.00		1.00	
1-90 TSDDs	1.05	(1.02 to 1.08)	1.04	(1.01 to 1.07)	1.05[b]	(0.98 to 1.11)
91-365 TSDDs	1.14	(1.07 to 1.21)	1.15	(1.07 to 1.24)		
366-1095 TSDDs	1.41	(1.27 to 1.56)	1.21	(1.06 to 1.38)		
>1095 TSDDs	1.08	(0.94 to 1.24)	1.02	(0.86 to 1.20)		
**Antiparkinson drugs**						
Non-use	1.00		1.00		1.00	
1-90 TSDDs	1.01	(0.73 to 1.39)	1.24	(0.89 to 1.73)	1.30[b]	(0.84 to 2.01)
91-365 TSDDs	1.68	(1.09 to 2.58)	1.54	(0.93 to 2.56)		
366-1095 TSDDs	1.03	(0.66 to 1.61)	1.29	(0.74 to 2.24)		
>1095 TSDDs	1.52	(1.16 to 2.00)	1.56	(1.16 to 2.11)		
**Antipsychotics**						
Non-use	1.00		1.00		1.00	
1-90 TSDDs	1.44	(1.25 to 1.66)	1.16	(0.99 to 1.36)	0.95	(0.70 to 1.27)
91-365 TSDDs	1.41	(1.21 to 1.65)	1.22	(1.03 to 1.45)	0.88	(0.62 to 1.24)
366-1095 TSDDs	2.09	(1.76 to 2.47)	1.64	(1.33 to 2.01)	1.37	(0.90 to 2.08)
>1095 TSDDs	1.70	(1.53 to 1.90)	1.42	(1.25 to 1.61)	1.23	(0.93 to 1.62)
**Bladder antimuscarinics**						
Non-use	1.00		1.00		1.00	
1-90 TSDDs	1.19	(1.13 to 1.26)	1.18	(1.11 to 1.26)	1.06	(0.91 to 1.23)
91-365 TSDDs	1.35	(1.27 to 1.45)	1.33	(1.22 to 1.44)	1.35	(1.12 to 1.63)
366-1095 TSDDs	1.65	(1.53 to 1.78)	1.55	(1.41 to 1.70)	1.48	(1.20 to 1.83)
>1095 TSDDs	1.65	(1.56 to 1.75)	1.57	(1.46 to 1.69)	1.55	(1.29 to 1.86)
**Skeletal muscle relaxants**						
Non-use	1.00		1.00		1.00	
1-90 TSDDs	0.98	(0.86 to 1.11)	1.01	(0.88 to 1.16)	1.24[b]	(0.95 to 1.61)
91-365 TSDDs	1.12	(0.77 to 1.65)	1.14	(0.72 to 1.80)		

© 2019 Coupland et al. JAMA Internal Medicine

366-1095 TSDDs	0.99	(0.46 to 2.10)	1.16	(0.50 to 2.68)		
>1095 TSDDs	1.10	(0.47 to 2.55)	1.03	(0.37 to 2.90)		
**Gastrointestinal antispasmodics**						
Non-use	1.00		1.00		1.00	
1-90 TSDDs	0.90	(0.85 to 0.94)	0.94	(0.89 to 0.99)	1.04[b]	(0.95 to 1.15)
91-365 TSDDs	0.93	(0.85 to 1.02)	1.03	(0.93 to 1.14)		
366-1095 TSDDs	0.93	(0.80 to 1.09)	0.98	(0.83 to 1.17)		
>1095 TSDDs	1.04	(0.90 to 1.20)	1.06	(0.90 to 1.26)		
**Antiarrhythmics**						
Non-use	1.00		1.00		1.00	
1-90 TSDDs	0.74	(0.33 to 1.64)	0.80	(0.34 to 1.90)	1.30[b]	(0.60 to 2.80)
91-365 TSDDs	1.25	(0.44 to 3.53)	1.17	(0.37 to 3.73)		
366-1095 TSDDs	1.22	(0.56 to 2.66)	0.97	(0.40 to 2.35)		
>1095 TSDDs	0.94	(0.56 to 1.55)	1.17	(0.70 to 1.96)		
**Antiepileptics**						
Non-use	1.00		1.00		1.00	
1-90 TSDDs	0.88	(0.80 to 0.97)	0.95	(0.86 to 1.06)	0.85	(0.68 to 1.08)
91-365 TSDDs	1.14	(0.95 to 1.36)	1.00	(0.82 to 1.21)	0.70	(0.45 to 1.11)
366-1095 TSDDs	1.13	(0.90 to 1.41)	1.19	(0.93 to 1.53)	0.69	(0.38 to 1.25)
>1095 TSDDs	1.39	(1.22 to 1.57)	1.37	(1.18 to 1.59)	1.66	(1.19 to 2.31)
**Antimuscarinic bronchodilators**						
Non-use	1.00		1.00		1.00	
1-90 TSDDs	0.99	(0.92 to 1.07)	0.97	(0.89 to 1.05)	0.88	(0.73 to 1.06)
91-365 TSDDs	0.97	(0.89 to 1.06)	1.02	(0.92 to 1.12)	0.85	(0.69 to 1.06)
366-1095 TSDDs	0.97	(0.88 to 1.06)	0.95	(0.86 to 1.06)	1.12	(0.86 to 1.45)
>1095 TSDDs	0.97	(0.90 to 1.05)	1.03	(0.94 to 1.13)	0.96	(0.78 to 1.19)

TSDD = total standardised daily dose

[a] Adjusted for other anticholinergic drug types, body mass index, smoking status, alcohol consumption, Townsend deprivation score, ethnic group, coronary heart disease, atrial fibrillation, heart failure, hypertension, hyperlipidaemia, diabetes (type 1 and type 2), stroke, transient ischaemic attack, subarachnoid haemorrhage, renal disease, asthma, chronic obstructive pulmonary disease, anxiety, depression, bipolar disorder, schizophrenia, severe head injury, cognitive decline/memory loss, antihypertensive drugs, aspirin, hypnotics, anxiolytic drugs, NSAIDs, statins and matching by age, sex, practice and calendar time.

[b] Any use of drug group (exposure categories combined into any use or no use due to small numbers)

© 2019 Coupland et al. JAMA Internal Medicine

eTable 7 Numbers of cases and controls prescribed anticholinergic drug types in the 1 to 11, 3 to 13 and 5 to 20 years before the index date

**Category**	**1 to 11 years before index date**		**3 to 13 years before index date**		**5 to 20 years before index date**	
	**Cases=58,769 Controls=225,574**		**Cases=45,621 Controls=169,020**		**Cases=8,283 Controls=27,200**	
	**Cases**	**Controls**	**Cases**	**Controls**	**Cases**	**Controls**
	**N (%)**	**N (%)**	**N (%)**	**N (%)**	**N (%)**	**N (%)**
**Antihistamines**						
Non-use	52312 (89.0)	202429 (89.7)	40678 (89.2)	151942 (89.9)	7083 (85.5)	23755 (87.3)
1-90 TSDDs	4987 (8.5)	18187 (8.1)	3890 (8.5)	13547 (8)	927 (11.2)	2730 (10)
91-365 TSDDs	923 (1.6)	3105 (1.4)	662 (1.5)	2268 (1.3)	189 (2.3)	484 (1.8)
366-1095 TSDDs	280 (0.5)	1022 (0.5)	216 (0.5)	727 (0.4)	51 (0.6)	127 (0.5)
>1095 TSDDs	267 (0.5)	831 (0.4)	175 (0.4)	536 (0.3)	33 (0.4)	104 (0.4)
**Antidepressants**						
Non-use	42831 (72.9)	173014 (76.7)	33396 (73.2)	130481 (77.2)	5771 (69.7)	20212 (74.3)
1-90 TSDDs	5098 (8.7)	19402 (8.6)	3903 (8.6)	13840 (8.2)	742 (9)	2239 (8.2)
91-365 TSDDs	3463 (5.9)	11931 (5.3)	2760 (6.1)	9142 (5.4)	600 (7.2)	1829 (6.7)
366-1095 TSDDs	2227 (3.8)	6749 (3.0)	1721 (3.8)	5209 (3.1)	386 (4.7)	1023 (3.8)
>1095 TSDDs	5150 (8.8)	14478 (6.4)	3841 (8.4)	10348 (6.1)	784 (9.5)	1897 (7)
**Antivertigo/ antiemetics**						
Non-use	44800 (76.2)	176584 (78.3)	35085 (76.9)	133102 (78.8)	6016 (72.6)	20337 (74.8)
1-90 TSDDs	11427 (19.4)	41159 (18.3)	8745 (19.2)	30489 (18)	1894 (22.9)	5874 (21.6)
91-365 TSDDs	1574 (2.7)	5026 (2.2)	1167 (2.6)	3526 (2.1)	247 (3)	634 (2.3)
366-1095 TSDDs	617 (1.1)	1659 (0.7)	382 (0.8)	1111 (0.7)	82 (1)	194 (0.7)
>1095 TSDDs	351 (0.6)	1146 (0.5)	242 (0.5)	792 (0.5)	44 (0.5)	161 (0.6)

© 2019 Coupland et al. JAMA Internal Medicine

**Antiparkinson drugs**						
Non-use	58477 (99.5)	225047 (99.8)	45399 (99.5)	168605 (99.8)	8235 (99.4)	27111 (99.7)
1-90 TSDDs	68 (0.1)	179 (0.1)	58 (0.1)	147 (0.1)	15 (0.2)	29 (0.1)
91-365 TSDDs	50 (0.1)	59 (0)	34 (0.1)	47 (0)	11 (0.1)	14 (0.1)
366-1095 TSDDs	39 (0.1)	71 (0)	27 (0.1)	51 (0)	6 (0.1)	11 (0)
>1095 TSDDs	135 (0.2)	218 (0.1)	103 (0.2)	170 (0.1)	16 (0.2)	35 (0.1)
**Antipsychotics**						
Non-use	56957 (96.9)	222174 (98.5)	44398 (97.3)	166299 (98.4)	8005 (96.6)	26516 (97.5)
1-90 TSDDs	388 (0.7)	882 (0.4)	269 (0.6)	737 (0.4)	73 (0.9)	225 (0.8)
91-365 TSDDs	332 (0.6)	695 (0.3)	238 (0.5)	593 (0.4)	48 (0.6)	162 (0.6)
366-1095 TSDDs	304 (0.5)	490 (0.2)	178 (0.4)	369 (0.2)	42 (0.5)	74 (0.3)
>1095 TSDDs	788 (1.3)	1333 (0.6)	538 (1.2)	1022 (0.6)	115 (1.4)	223 (0.8)
**Bladder antimuscarinics**						
Non-use	51905 (88.3)	206796 (91.7)	41043 (90.0)	156668 (92.7)	7452 (90)	25178 (92.6)
1-90 TSDDs	2139 (3.6)	7005 (3.1)	1515 (3.3)	4798 (2.8)	281 (3.4)	834 (3.1)
91-365 TSDDs	1417 (2.4)	4078 (1.8)	1030 (2.3)	2834 (1.7)	195 (2.4)	441 (1.6)
366-1095 TSDDs	1244 (2.1)	2941 (1.3)	789 (1.7)	1855 (1.1)	149 (1.8)	309 (1.1)
>1095 TSDDs	2064 (3.5)	4754 (2.1)	1244 (2.7)	2865 (1.7)	206 (2.5)	438 (1.6)
**Skeletal muscle relaxants**						
Non-use	58340 (99.3)	224006 (99.3)	45266 (99.2)	167796 (99.3)	8186 (98.8)	26949 (99.1)
1-90 TSDDs	372 (0.6)	1380 (0.6)	315 (0.7)	1089 (0.6)	86 (1.0)	228 (0.8)
91-365 TSDDs	39 (0.1)	115 (0.1)	27 (0.1)	88 (0.1)	9 (0.1)	16 (0.1)
366-1095 TSDDs	9 (0)	41 (0)	8 (0)	24 (0)	≤5 (0)	6 (0)
>1095 TSDDs	9 (0)	32 (0)	≤5 (0)	23 (0)	≤5 (0)	≤5 (0)
**Gastrointestinal antispasmodics**						
Non-use	54733 (93.1)	210093 (93.1)	42416 (93)	157655 (93.3)	7496 (90.5)	24900 (91.5)

© 2019 Coupland et al. JAMA Internal Medicine

1-90 TSDDs	2765 (4.7)	10914 (4.8)	2192 (4.8)	8015 (4.7)	565 (6.8)	1656 (6.1)
91-365 TSDDs	722 (1.2)	2686 (1.2)	592 (1.3)	1997 (1.2)	137 (1.7)	396 (1.5)
366-1095 TSDDs	267 (0.5)	938 (0.4)	217 (0.5)	695 (0.4)	42 (0.5)	138 (0.5)
>1095 TSDDs	282 (0.5)	943 (0.4)	204 (0.5)	658 (0.4)	43 (0.5)	110 (0.4)
**Antiarrhythmic drugs**						
Non-use	58720 (99.9)	225402 (99.9)	45580 (99.9)	168877 (99.9)	8272 (99.9)	27175 (99.9)
1-90 TSDDs	9 (0)	37 (0)	7 (0)	32 (0)	≤5 (0)	8 (0)
91-365 TSDDs	7 (0)	20 (0)	≤5 (0)	17 (0)	≤5 (0)	≤5 (0)
366-1095 TSDDs	10 (0)	27 (0)	7 (0)	24 (0)	≤5 (0)	≤5 (0)
>1095 TSDDs	23 (0)	88 (0)	23 (0.1)	70 (0)	6 (0.1)	14 (0.1)
**Antiepileptics**						
Non-use	57358 (97.6)	221082 (98)	44481 (97.5)	165612 (98)	117 (1.4)	26516 (97.5)
1-90 TSDDs	630 (1.1)	2459 (1.1)	549 (1.2)	1864 (1.1)	30 (0.4)	392 (1.4)
91-365 TSDDs	202 (0.3)	592 (0.3)	167 (0.4)	504 (0.3)	15 (0.2)	107 (0.4)
366-1095 TSDDs	135 (0.2)	359 (0.2)	106 (0.2)	274 (0.2)	10 (0.1)	65 (0.2)
>1095 TSDDs	444 (0.8)	1082 (0.5)	318 (0.7)	766 (0.5)	66 (0.8)	120 (0.4)
**Antimuscarinic bronchodilators**						
Non-use	54891 (93.4)	211578 (93.8)	42699 (93.6)	159021 (94.1)	185 (2.2)	25530 (93.9)
1-90 TSDDs	1228 (2.1)	4326 (1.9)	921 (2)	3172 (1.9)	124 (1.5)	588 (2.2)
91-365 TSDDs	786 (1.3)	2885 (1.3)	628 (1.4)	2135 (1.3)	90 (1.1)	399 (1.5)
366-1095 TSDDs	742 (1.3)	2719 (1.2)	542 (1.2)	1925 (1.1)	39 (0.5)	246 (0.9)
>1095 TSDDs	1122 (1.9)	4066 (1.8)	831 (1.8)	2767 (1.6)	139 (1.7)	437 (1.6)

© 2019 Coupland et al. JAMA Internal Medicine

eTable 8 Adjusted odds ratios for cumulative use of anticholinergic drugs in the 1 to 11 years before index date: separate analyses in cases aged less than 80 at diagnosis of dementia and cases aged 80 and over at diagnosis, with their matched controls

**Exposure category**	**Aged <80 at index date: Cases= 18,302 Controls= 72,000**		**Aged ≥80 at index date: Cases= 40,467 Controls= 153,574**	
	**Adjusted[a]**		**Adjusted[a]**	
	**OR**	**95% CI**	**OR**	**95% CI**
**Cumulative use: all drugs**				
Non-use	1.00		1.00	
1-90 TSDDs	1.07	(1.02 to 1.13)	1.05	(1.02 to 1.08)
91-365 TSDDs	1.32	(1.24 to 1.41)	1.10	(1.05 to 1.14)
366-1095 TSDDs	1.53	(1.42 to 1.65)	1.28	(1.22 to 1.35)
>1095 TSDDs	1.81	(1.71 to 1.91)	1.35	(1.30 to 1.40)
**Antihistamines**				
Non-use	1.00		1.00	
1-90 TSDDs	1.11	(1.04 to 1.18)	0.99	(0.95 to 1.03)
91-365 TSDDs	0.99	(0.85 to 1.16)	1.04	(0.94 to 1.14)
366-1095 TSDDs	0.98	(0.74 to 1.30)	1.02	(0.86 to 1.21)
>1095 TSDDs	1.41	(1.08 to 1.86)	1.02	(0.84 to 1.24)
**Antidepressants**				
Non-use	1.00		1.00	
1-90 TSDDs	1.04	(0.97 to 1.11)	1.01	(0.96 to 1.05)
91-365 TSDDs	1.27	(1.17 to 1.37)	1.06	(1.00 to 1.12)
366-1095 TSDDs	1.34	(1.21 to 1.47)	1.21	(1.13 to 1.29)
>1095 TSDDs	1.47	(1.37 to 1.57)	1.20	(1.14 to 1.26)
**Antivertigo/antiemetics**				
Non-use	1.00		1.00	
1-90 TSDDs	1.10	(1.05 to 1.16)	1.03	(0.99 to 1.06)
91-365 TSDDs	1.25	(1.10 to 1.41)	1.10	(1.02 to 1.19)
366-1095 TSDDs	1.72	(1.39 to 2.13)	1.33	(1.18 to 1.50)
>1095 TSDDs	1.27	(0.97 to 1.67)	1.02	(0.87 to 1.19)
**Antiparkinson agents**				
Non-use	1.00		1.00	
1-90 TSDDs	1.24	(0.75 to 2.07)	0.86	(0.56 to 1.32)
91-365 TSDDs	1.91	(1.06 to 3.45)	1.31	(0.68 to 2.50)
366-1095 TSDDs	1.19	(0.62 to 2.26)	0.89	(0.46 to 1.71)
>1095 TSDDs	1.70	(1.18 to 2.45)	1.21	(0.80 to 1.84)
**Antipsychotics**				
Non-use	1.00		1.00	
1-90 TSDDs	1.32	(1.03 to 1.7)	1.48	(1.25 to 1.75)
91-365 TSDDs	1.58	(1.22 to 2.06)	1.32	(1.09 to 1.60)
366-1095 TSDDs	2.74	(2.05 to 3.66)	1.82	(1.48 to 2.25)
>1095 TSDDs	2.13	(1.79 to 2.53)	1.44	(1.25 to 1.66)

© 2019 Coupland et al. JAMA Internal Medicine

**Bladder antimuscarinics**				
Non-use	1.00		1.00	
1-90 TSDDs	1.22	(1.10 to 1.37)	1.19	(1.12 to 1.27)
91-365 TSDDs	1.46	(1.28 to 1.66)	1.32	(1.22 to 1.43)
366-1095 TSDDs	1.91	(1.66 to 2.20)	1.55	(1.42 to 1.70)
>1095 TSDDs	1.94	(1.73 to 2.17)	1.56	(1.46 to 1.67)
**Skeletal muscle relaxants**				
Non-use	1.00		1.00	
1-90 TSDDs	0.86	(0.69 to 1.06)	1.05	(0.90 to 1.24)
91-365 TSDDs	1.39	(0.82 to 2.33)	0.85	(0.47 to 1.51)
366-1095 TSDDs	1.07	(0.41 to 2.82)	0.78	(0.22 to 2.75)
>1095 TSDDs	1.27	(0.44 to 3.69)	0.68	(0.15 to 3.14)
**Gastrointestinal antispasmodics**				
Non-use	1.00		1.00	
1-90 TSDDs	0.92	(0.85 to 1.00)	0.88	(0.83 to 0.94)
91-365 TSDDs	0.97	(0.83 to 1.14)	0.91	(0.82 to 1.03)
366-1095 TSDDs	0.90	(0.70 to 1.16)	0.94	(0.78 to 1.14)
>1095 TSDDs	1.06	(0.84 to 1.35)	1.01	(0.84 to 1.21)
**Antiarrhythmic drugs**				
Non-use	1.00		1.00	
1-90 TSDDs	1.58	(0.54 to 4.63)	0.39	(0.11 to 1.33)
91-365 TSDDs	1.03	(0.27 to 3.95)	1.53	(0.29 to 8.07)
366-1095 TSDDs	1.19	(0.32 to 4.44)	1.24	(0.47 to 3.28)
>1095 TSDDs	0.55	(0.18 to 1.67)	1.06	(0.60 to 1.87)
**Antiepileptics**				
Non-use	1.00		1.00	
1-90 TSDDs	0.90	(0.75 to 1.08)	0.87	(0.78 to 0.98)
91-365 TSDDs	1.03	(0.75 to 1.40)	1.20	(0.96 to 1.49)
366-1095 TSDDs	1.15	(0.79 to 1.66)	1.09	(0.82 to 1.45)
>1095 TSDDs	1.51	(1.24 to 1.85)	1.26	(1.07 to 1.49)
**Antimuscarinic bronchodilators**				
Non-use	1.00		1.00	
1-90 TSDDs	1.07	(0.94 to 1.22)	0.96	(0.88 to 1.05)
91-365 TSDDs	1.03	(0.88 to 1.20)	0.93	(0.84 to 1.04)
366-1095 TSDDs	0.97	(0.82 to 1.16)	0.97	(0.87 to 1.08)
>1095 TSDDs	1.02	(0.89 to 1.18)	0.94	(0.86 to 1.04)

TSDD = total standardised daily dose

[a] Adjusted for body mass index, smoking status, alcohol consumption, Townsend deprivation score, ethnic group, coronary heart disease, atrial fibrillation, heart failure, hypertension, hyperlipidaemia, diabetes (type 1 and type 2), stroke, transient ischaemic attack, subarachnoid haemorrhage, renal disease, asthma, chronic obstructive pulmonary disease, anxiety, depression, bipolar disorder, schizophrenia, severe head injury, cognitive decline/memory loss, antihypertensive drugs, aspirin, hypnotics, anxiolytic drugs, NSAIDs, statins and with matching by age, sex, practice and calendar time. Individual types of anticholinergic drug are also adjusted for the other anticholinergic drug types.

© 2019 Coupland et al. JAMA Internal Medicine

eTable 9 Adjusted odds ratios for cumulative use of anticholinergic drugs in the 1 to 11 years before index date: separate analyses in men and women

Category	Men: Cases= 21,664 Controls= 83,314		Women: Cases= 37,105 Controls= 142,260	
	Adjusted[a]		Adjusted[a]	
	OR	95% CI	OR	95% CI
**Cumulative use: all drugs**				
Non-use	1.00		1.00	
1-90 TSDDs	1.11	(1.06 to 1.16)	1.03	(1.00 to 1.07)
91-365 TSDDs	1.26	(1.19 to 1.34)	1.12	(1.07 to 1.16)
366-1095 TSDDs	1.44	(1.35 to 1.55)	1.31	(1.24 to 1.37)
>1095 TSDDs	1.58	(1.49 to 1.67)	1.44	(1.39 to 1.50)
**Antihistamines**				
Non-use	1.00		1.00	
1-90 TSDDs	1.08	(1.01 to 1.15)	1.00	(0.96 to 1.05)
91-365 TSDDs	1.13	(0.98 to 1.30)	0.97	(0.88 to 1.08)
366-1095 TSDDs	0.90	(0.70 to 1.16)	1.08	(0.91 to 1.29)
>1095 TSDDs	1.12	(0.83 to 1.50)	1.16	(0.96 to 1.39)
**Antidepressants**				
Non-use	1.00		1.00	
1-90 TSDDs	1.01	(0.95 to 1.08)	1.02	(0.97 to 1.06)
91-365 TSDDs	1.09	(1.00 to 1.18)	1.13	(1.07 to 1.19)
366-1095 TSDDs	1.25	(1.12 to 1.39)	1.25	(1.17 to 1.33)
>1095 TSDDs	1.33	(1.23 to 1.44)	1.28	(1.22 to 1.34)
**Antivertigo/antiemetics**				
Non-use	1.00		1.00	
1-90 TSDDs	1.11	(1.06 to 1.16)	1.02	(0.99 to 1.06)
91-365 TSDDs	1.26	(1.11 to 1.44)	1.10	(1.02 to 1.18)
366-1095 TSDDs	1.48	(1.19 to 1.83)	1.39	(1.23 to 1.56)
>1095 TSDDs	1.12	(0.84 to 1.50)	1.07	(0.92 to 1.25)
**Antiparkinson agents**				
Non-use	1.00		1.00	
1-90 TSDDs	1.15	(0.66 to 1.99)	0.92	(0.62 to 1.36)
91-365 TSDDs	1.67	(0.79 to 3.52)	1.65	(0.98 to 2.79)
366-1095 TSDDs	1.85	(0.87 to 3.94)	0.73	(0.40 to 1.30)
>1095 TSDDs	1.36	(0.83 to 2.23)	1.57	(1.14 to 2.17)
**Antipsychotics**				
Non-use	1.00		1.00	
1-90 TSDDs	1.48	(1.16 to 1.89)	1.42	(1.20 to 1.69)
91-365 TSDDs	1.57	(1.20 to 2.07)	1.34	(1.11 to 1.61)
366-1095 TSDDs	2.39	(1.73 to 3.31)	2.00	(1.64 to 2.43)
>1095 TSDDs	2.01	(1.62 to 2.50)	1.60	(1.41 to 1.82)
**Bladder antimuscarinics**				
Non-use	1.00		1.00	
1-90 TSDDs	1.12	(1.02 to 1.24)	1.23	(1.15 to 1.32)

© 2019 Coupland et al. JAMA Internal Medicine

91-365 TSDDs	1.52	(1.36 to 1.70)	1.27	(1.17 to 1.39)
366-1095 TSDDs	1.75	(1.54 to 1.98)	1.60	(1.46 to 1.75)
>1095 TSDDs	1.76	(1.58 to 1.95)	1.61	(1.50 to 1.73)
**Skeletal muscle relaxants**				
Non-use	1.00		1.00	
1-90 TSDDs	0.94	(0.76 to 1.16)	1.00	(0.85 to 1.18)
91-365 TSDDs	1.01	(0.53 to 1.93)	1.17	(0.73 to 1.88)
366-1095 TSDDs	0.90	(0.29 to 2.81)	1.04	(0.38 to 2.89)
>1095 TSDDs	1.36	(0.41 to 4.55)	0.92	(0.28 to 2.95)
**Gastrointestinal antispasmodics**				
Non-use	1.00		1.00	
1-90 TSDDs	0.90	(0.83 to 0.98)	0.89	(0.84 to 0.94)
91-365 TSDDs	0.93	(0.77 to 1.11)	0.94	(0.84 to 1.04)
366-1095 TSDDs	0.91	(0.66 to 1.25)	0.95	(0.80 to 1.12)
>1095 TSDDs	1.09	(0.81 to 1.47)	1.03	(0.87 to 1.22)
**Antiarrhythmic drugs**				
Non-use	1.00		1.00	
1-90 TSDDs	0.35	(0.04 to 2.95)	0.85	(0.36 to 2.03)
91-365 TSDDs	0.58	(0.07 to 5.01)	1.78	(0.52 to 6.05)
366-1095 TSDDs	1.33	(0.31 to 5.65)	1.19	(0.47 to 3.01)
>1095 TSDDs	1.93	(0.74 to 5.00)	0.73	(0.40 to 1.35)
**Antiepileptics**				
Non-use	1.00		1.00	
1-90 TSDDs	0.90	(0.75 to 1.07)	0.87	(0.77 to 0.98)
91-365 TSDDs	1.45	(1.09 to 1.93)	0.99	(0.79 to 1.25)
366-1095 TSDDs	1.19	(0.83 to 1.72)	1.09	(0.83 to 1.45)
>1095 TSDDs	1.47	(1.21 to 1.78)	1.34	(1.13 to 1.58)
**Antimuscarinic bronchodilators**				
Non-use	1.00		1.00	
1-90 TSDDs	1.03	(0.92 to 1.15)	0.97	(0.88 to 1.06)
91-365 TSDDs	1.03	(0.90 to 1.17)	0.92	(0.81 to 1.03)
366-1095 TSDDs	1.10	(0.96 to 1.26)	0.88	(0.77 to 1.00)
>1095 TSDDs	0.95	(0.84 to 1.07)	0.98	(0.88 to 1.09)

TSDD = total standardised daily dose

[a] Adjusted for body mass index, smoking status, alcohol consumption, Townsend deprivation score, ethnic group, coronary heart disease, atrial fibrillation, heart failure, hypertension, hyperlipidaemia, diabetes (type 1 and type 2), stroke, transient ischaemic attack, subarachnoid haemorrhage, renal disease, asthma, chronic obstructive pulmonary disease, anxiety, depression, bipolar disorder, schizophrenia, severe head injury, cognitive decline/memory loss, antihypertensive drugs, aspirin, hypnotics, anxiolytic drugs, NSAIDs, statins and with matching by age, sex, practice and calendar time. Individual types of anticholinergic drug are also adjusted for the other anticholinergic drug types.

© 2019 Coupland et al. JAMA Internal Medicine

eTable 10 Adjusted odds ratios for cumulative use of anticholinergic drugs in the 1 to 11 years before index date: separate analyses in cases with Alzheimer's (including mixed), cases with vascular dementia, and cases with other or unspecified types of dementia with their respective matched controls

Category	Alzheimer's		Vascular dementia		Other/unspecified dementia types	
	Cases=22,034 Controls=85,595		Cases=13,313 Controls=51,535		Cases=23,422 Controls=88,444	
	Adjusted[a]		Adjusted[a]		Adjusted[a]	
	OR	95% CI	OR	95% CI	OR	95% CI
**Cumulative use: all drugs**						
Non-use	1.00		1.00		1.00	
1-90 TSDDs	1.05	(1.01 to 1.10)	1.09	(1.03 to 1.16)	1.05	(1.01 to 1.10)
91-365 TSDDs	1.11	(1.05 to 1.18)	1.22	(1.14 to 1.32)	1.18	(1.12 to 1.25)
366-1095 TSDDs	1.27	(1.19 to 1.36)	1.54	(1.41 to 1.67)	1.34	(1.26 to 1.43)
>1095 TSDDs	1.37	(1.30 to 1.44)	1.68	(1.57 to 1.79)	1.51	(1.44 to 1.59)
**Antihistamines**						
Non-use	1.00		1.00		1.00	
1-90 TSDDs	1.05	(0.99 to 1.12)	1.04	(0.96 to 1.12)	0.99	(0.93 to 1.05)
91-365 TSDDs	0.99	(0.86 to 1.14)	0.99	(0.83 to 1.18)	1.10	(0.96 to 1.25)
366-1095 TSDDs	1.04	(0.82 to 1.32)	1.24	(0.92 to 1.68)	0.90	(0.71 to 1.14)
>1095 TSDDs	1.23	(0.97 to 1.58)	0.93	(0.65 to 1.34)	1.21	(0.94 to 1.54)
**Antidepressants**						
Non-use	1.00		1.00		1.00	
1-90 TSDDs	1.01	(0.95 to 1.07)	1.04	(0.97 to 1.13)	1.01	(0.95 to 1.07)
91-365 TSDDs	1.11	(1.03 to 1.19)	1.14	(1.04 to 1.25)	1.13	(1.06 to 1.22)
366-1095 TSDDs	1.19	(1.09 to 1.30)	1.29	(1.15 to 1.45)	1.28	(1.17 to 1.40)
>1095 TSDDs	1.30	(1.22 to 1.39)	1.37	(1.27 to 1.49)	1.24	(1.16 to 1.32)
**Antivertigo/antiemetics**						
Non-use	1.00		1.00		1.00	
1-90 TSDDs	1.03	(0.99 to 1.08)	1.10	(1.04 to 1.16)	1.04	(1.00 to 1.09)
91-365 TSDDs	1.12	(1.01 to 1.25)	1.22	(1.07 to 1.40)	1.11	(1.00 to 1.23)
366-1095 TSDDs	1.29	(1.08 to 1.54)	1.49	(1.20 to 1.85)	1.49	(1.26 to 1.76)
>1095 TSDDs	0.91	(0.72 to 1.16)	1.11	(0.84 to 1.47)	1.24	(1.01 to 1.53)
**Antiparkinson drugs**						
Non-use	1.00		1.00		1.00	
1-90 TSDDs	0.71	(0.37 to 1.36)	1.00	(0.52 to 1.91)	1.31	(0.82 to 2.08)
91-365 TSDDs	1.12	(0.45 to 2.81)	0.82	(0.30 to 2.21)	2.46	(1.36 to 4.45)
366-1095 TSDDs	0.51	(0.19 to 1.33)	1.46	(0.55 to 3.84)	1.20	(0.65 to 2.23)
>1095 TSDDs	1.16	(0.70 to 1.93)	1.76	(0.95 to 3.28)	1.72	(1.17 to 2.53)
**Antipsychotics**						
Non-use	1.00		1.00		1.00	
1-90 TSDDs	1.18	(0.91 to 1.52)	1.68	(1.27 to 2.24)	1.49	(1.21 to 1.85)
91-365 TSDDs	0.98	(0.74 to 1.29)	1.61	(1.16 to 2.22)	1.72	(1.37 to 2.17)
366-1095 TSDDs	1.29	(0.94 to 1.76)	2.13	(1.49 to 3.07)	2.84	(2.22 to 3.65)
>1095 TSDDs	1.32	(1.08 to 1.62)	1.59	(1.26 to 2.01)	2.11	(1.79 to 2.47)

© 2019 Coupland et al. JAMA Internal Medicine

**Bladder antimuscarinics**						
Non-use	1.00		1.00		1.00	
1-90 TSDDs	1.26	(1.16 to 1.38)	1.22	(1.09 to 1.36)	1.10	(1.00 to 1.21)
91-365 TSDDs	1.20	(1.07 to 1.34)	1.57	(1.37 to 1.80)	1.37	(1.23 to 1.54)
366-1095 TSDDs	1.57	(1.39 to 1.78)	1.80	(1.54 to 2.09)	1.62	(1.44 to 1.83)
>1095 TSDDs	1.58	(1.43 to 1.73)	1.89	(1.69 to 2.13)	1.58	(1.44 to 1.75)
**Skeletal muscle relaxants**						
Non-use	1.00		1.00		1.00	
Any use	1.09[†]	(0.90 to 1.32)	0.91[†]	(0.71 to 1.16)	0.94[†]	(0.77 to 1.15)
**Gastrointestinal antispasmodics**						
Non-use	1.00		1.00		1.00	
1-90 TSDDs	0.89	(0.83 to 0.96)	0.91	(0.83 to 1.01)	0.89	(0.82 to 0.96)
91-365 TSDDs	0.98	(0.85 to 1.13)	1.02	(0.84 to 1.24)	0.84	(0.71 to 0.98)
366-1095 TSDDs	0.93	(0.74 to 1.19)	0.98	(0.71 to 1.35)	0.92	(0.71 to 1.18)
>1095 TSDDs	0.89	(0.70 to 1.13)	1.23	(0.93 to 1.63)	1.09	(0.85 to 1.40)
**Antiarrhythmic drugs**						
Non-use	1.00		1.00		1.00	
Any use	1.27[†]	(0.74 to 2.17)	1.15[†]	(0.55 to 2.39)	0.67[†]	(0.36 to 1.23)
**Antiepileptic drugs**						
Non-use	1.00		1.00		1.00	
1-90 TSDDs	1.22	(0.42 to 3.50)	1.04	(0.85 to 1.27)	0.86	(0.74 to 1.01)
91-365 TSDDs	1.11	(0.23 to 5.40)	1.27	(0.90 to 1.80)	1.16	(0.87 to 1.55)
366-1095 TSDDs	2.41	(0.75 to 7.73)	1.34	(0.87 to 2.06)	0.98	(0.67 to 1.43)
>1095 TSDDs	0.99	(0.42 to 2.34)	1.96	(1.55 to 2.49)	1.34	(1.09 to 1.66)
**Antimuscarinic bronchodilators**						
Non-use	1.00		1.00		1.00	
1-90 TSDDs	0.81	(0.69 to 0.96)	1.12	(0.98 to 1.30)	1.02	(0.9 to 1.14)
91-365 TSDDs	1.02	(0.76 to 1.39)	1.03	(0.86 to 1.23)	0.95	(0.82 to 1.10)
366-1095 TSDDs	1.15	(0.80 to 1.66)	1.18	(0.99 to 1.41)	0.97	(0.84 to 1.13)
>1095 TSDDs	1.12	(0.90 to 1.39)	0.99	(0.84 to 1.16)	1.00	(0.88 to 1.13)

[†] Any use of drug group (categories combined due to small numbers)

TSDD = total standardised daily dose

[a] Adjusted for body mass index, smoking status, alcohol consumption, Townsend deprivation score, ethnic group, coronary heart disease, atrial fibrillation, heart failure, hypertension, hyperlipidaemia, diabetes (type 1 and type 2), stroke, transient ischaemic attack, subarachnoid haemorrhage, renal disease, asthma, chronic obstructive pulmonary disease, anxiety, depression, bipolar disorder, schizophrenia, severe head injury, cognitive decline/memory loss, antihypertensive drugs, aspirin, hypnotics, anxiolytic drugs, NSAIDs, statins and with matching by age, sex, practice and calendar time. Individual types of anticholinergic drug are also adjusted for the other anticholinergic drug types.

© 2019 Coupland et al. JAMA Internal Medicine

eTable 11 Adjusted odds ratios for total cumulative use in defined daily doses (DDDs) of anticholinergic drugs in 1 to 11, 3 to 13 and 5 to 20 years before the index date

Cumulative use (total DDDs)	Cases	Controls	Adjusted[a]	
**Category**	**n (%)**	**n (%)**	**OR**	**95% CI**
**Exposure in 1 to 11 years before index date**				
	N=58,769	N=225,574		
Non-use	25516 (43.4)	110478 (49.0)	1.00	
1-90 DDDs	18239 (31.0)	70614 (31.3)	1.10	(1.07 to 1.12)
91-365 DDDs	5272 (9.0)	17002 (7.5)	1.31	(1.26 to 1.36)
366-1095 DDDs	4402 (7.5)	12826 (5.7)	1.43	(1.37 to 1.49)
>1095 DDDs	5340 (9.1)	14654 (6.5)	1.52	(1.46 to 1.58)
**Exposure in 3 to 13 years before index date**				
	N=45,621	N=169,020		
Non-use	20545 (45.0)	84676 (50.1)	1.00	
1-90 DDDs	14191 (31.1)	52954 (31.3)	1.08	(1.05 to 1.11)
91-365 DDDs	4008 (8.8)	12434 (7.4)	1.29	(1.23 to 1.35)
366-1095 DDDs	3121 (6.8)	8979 (5.3)	1.37	(1.30 to 1.44)
>1095 DDDs	3756 (8.2)	9977 (5.9)	1.50	(1.43 to 1.57)
**Exposure in 5 to 20 years before index date**				
	N=8,283	N= 27,200		
Non-use	3335 (40.3)	12281 (45.2)	1.00	
1-90 DDDs	2784 (33.6)	9416 (34.6)	1.07	(1.01 to 1.14)
91-365 DDDs	844 (10.2)	2260 (8.3)	1.29	(1.17 to 1.42)
366-1095 DDDs	593 (7.2)	1439 (5.3)	1.47	(1.31 to 1.65)
>1095 DDDs	727 (8.8)	1804 (6.6)	1.46	(1.31 to 1.63)

DDD = defined daily dose (WHO)

[a] Adjusted for body mass index, smoking status, alcohol consumption, Townsend deprivation score, ethnic group, coronary heart disease, atrial fibrillation, heart failure, hypertension, hyperlipidaemia, diabetes (type 1 and type 2), stroke, transient ischaemic attack, subarachnoid haemorrhage, renal disease, asthma, chronic obstructive pulmonary disease, anxiety, depression, bipolar disorder, schizophrenia, severe head injury, cognitive decline/memory loss, antihypertensive drugs, aspirin, hypnotics, anxiolytic drugs, NSAIDs, statins and with matching by age, sex, practice and calendar time.

© 2019 Coupland et al. JAMA Internal Medicine

eTable 12 Adjusted odds ratios for cumulative use of anticholinergic drugs in 1 to 11 years before index date: multiple imputation results

	Complete case analysis		Multiple imputation analysis	
**Category**	**Adjusted[a]**		**Adjusted[a]**	
	**OR**	**95% CI**	**OR**	**95% CI**
**Cumulative use (TSDDs)**				
Non-use	1.00		1.00	
1-90 TSDDs	1.06	(1.03 to 1.09)	1.07	(1.04 to 1.10)
91-365 TSDDs	1.17	(1.13 to 1.21)	1.17	(1.13 to 1.21)
366-1095 TSDDs	1.36	(1.30 to 1.41)	1.35	(1.30 to 1.41)
>1095 TSDDs	1.49	(1.44 to 1.54)	1.49	(1.44 to 1.53)
**Cumulative use (total DDDs)**				
Non-use	1.00		1.00	
1-90 DDDs	1.10	(1.07 to 1.12)	1.10	(1.08 to 1.13)
91-365 DDDs	1.31	(1.26 to 1.36)	1.31	(1.26 to 1.35)
366-1095 DDDs	1.43	(1.37 to 1.49)	1.43	(1.38 to 1.49)
>1095 DDDs	1.52	(1.46 to 1.58)	1.51	(1.46 to 1.57)

TSDD = total standardised daily dose
DDD = defined daily dose
[a] Adjusted for body mass index, smoking status, alcohol consumption, Townsend deprivation score, ethnic group, coronary heart disease, atrial fibrillation, heart failure, hypertension, hyperlipidaemia, diabetes (type 1 and type 2), stroke, transient ischaemic attack, subarachnoid haemorrhage, renal disease, asthma, chronic obstructive pulmonary disease, anxiety, depression, bipolar disorder, schizophrenia, severe head injury, cognitive decline/memory loss, antihypertensive drugs, aspirin, hypnotics, anxiolytic drugs, NSAIDs, statins and with matching by age, sex, practice and calendar time.

© 2019 Coupland et al. JAMA Internal Medicine

eTable 13 Adjusted odds ratios for cumulative use of anticholinergic drugs in the 1 to 11 years before index date: restricted to the anticholinergic drugs included in the study by Gray[1]

Category	Cases (N=58,769)	Controls (N=225,574)	Unadjusted		Adjusted[a]	
	N (%)	N (%)	OR	95% CI	OR	95% CI
**Cumulative use: all drugs**						
Non-use	28390 (48.3)	120310 (53.3)	1.00		1.00	
1-90 TSDDs	13149 (22.4)	52200 (23.1)	1.07	(1.05 to 1.10)	1.05	(1.02 to 1.08)
91-365 TSDDs	5984 (10.2)	21477 (9.5)	1.19	(1.16 to 1.23)	1.16	(1.12 to 1.20)
366-1095 TSDDs	3791 (6.5)	11304 (5.0)	1.43	(1.38 to 1.49)	1.39	(1.33 to 1.45)
>1095 TSDDs	7455 (12.7)	20283 (9.0)	1.58	(1.53 to 1.63)	1.49	(1.44 to 1.55)
**Antihistamines**						
Non-use	52312 (89)	202429 (89.7)	1.00		1.00	
1-90 TSDDs	4987 (8.5)	18187 (8.1)	1.02	(0.99 to 1.06)	1.03	(0.99 to 1.07)
91-365 TSDDs	923 (1.6)	3105 (1.4)	1.07	(0.99 to 1.16)	1.03	(0.95 to 1.12)
366-1095 TSDDs	280 (0.5)	1022 (0.5)	0.98	(0.86 to 1.13)	1.02	(0.88 to 1.18)
>1095 TSDDs	267 (0.5)	831 (0.4)	1.15	(0.99 to 1.32)	1.15	(0.98 to 1.34)
**Antidepressants**						
Non-use	45097 (76.7)	179412 (79.5)	1.00		1.00	
1-90 TSDDs	4566 (7.8)	17760 (7.9)	1.00	(0.97 to 1.04)	0.98	(0.94 to 1.02)
91-365 TSDDs	3058 (5.2)	10786 (4.8)	1.10	(1.06 to 1.15)	1.07	(1.02 to 1.12)
366-1095 TSDDs	1822 (3.1)	5609 (2.5)	1.23	(1.17 to 1.30)	1.21	(1.14 to 1.28)
>1095 TSDDs	4226 (7.2)	12007 (5.3)	1.33	(1.28 to 1.38)	1.26	(1.21 to 1.31)
**Antivertigo/antiemetic**						
Non-use	44800 (76.2)	176584 (78.3)	1.00		1.00	
1-90 TSDDs	11427 (19.4)	41159 (18.3)	1.07	(1.04 to 1.09)	1.05	(1.02 to 1.08)
91-365 TSDDs	1574 (2.7)	5026 (2.2)	1.16	(1.09 to 1.23)	1.15	(1.08 to 1.23)
366-1095 TSDDs	617 (1.1)	1659 (0.7)	1.37	(1.24 to 1.50)	1.43	(1.28 to 1.58)
>1095 TSDDs	351 (0.6)	1146 (0.5)	1.09	(0.96 to 1.23)	1.10	(0.96 to 1.26)
**Antiparkinson agents**						
Non-use	58477 (99.5)	225047 (99.8)	1.00		1.00	
1-90 TSDDs	68 (0.1)	179 (0.1)	1.09	(0.81 to 1.45)	1.04	(0.75 to 1.43)
91-365 TSDDs	50 (0.1)	59 (0)	2.19	(1.48 to 3.24)	1.76	(1.15 to 2.71)
366-1095 TSDDs	39 (0.1)	71 (0)	1.41	(0.94 to 2.12)	1.11	(0.71 to 1.74)
>1095 TSDDs	135 (0.2)	218 (0.1)	1.66	(1.32 to 2.09)	1.55	(1.18 to 2.03)
**Antipsychotics**						
Non-use	57432 (97.7)	222927 (98.8)	1.00		1.00	
1-90 TSDDs	270 (0.5)	663 (0.3)	1.48	(1.28 to 1.72)	1.39	(1.18 to 1.64)
91-365 TSDDs	246 (0.4)	591 (0.3)	1.47	(1.26 to 1.71)	1.23	(1.04 to 1.47)
366-1095 TSDDs	218 (0.4)	372 (0.2)	2.11	(1.78 to 2.50)	2.08	(1.72 to 2.53)
>1095 TSDDs	603 (1.0)	1021 (0.5)	1.90	(1.71 to 2.12)	1.68	(1.48 to 1.91)
**Bladder antimuscarinics**						
Non-use	51933 (88.4)	206879 (91.7)	1.00		1.00	

© 2019 Coupland et al. JAMA Internal Medicine

1-90 TSDDs	2144 (3.7)	7008 (3.1)	1.19	(1.13 to 1.25)	1.20	(1.14 to 1.27)
91-365 TSDDs	1420 (2.4)	4069 (1.8)	1.34	(1.26 to 1.43)	1.37	(1.28 to 1.46)
366-1095 TSDDs	1251 (2.1)	2935 (1.3)	1.65	(1.54 to 1.77)	1.67	(1.55 to 1.80)
>1095 TSDDs	2021 (3.4)	4683 (2.1)	1.65	(1.57 to 1.75)	1.65	(1.55 to 1.75)
**Gastrointestinal antispasmodics**						
Non-use	54733 (93.1)	210093 (93.1)	1.00		1.00	
1-90 TSDDs	2765 (4.7)	10914 (4.8)	0.91	(0.87 to 0.95)	0.90	(0.86 to 0.94)
91-365 TSDDs	722 (1.2)	2686 (1.2)	0.95	(0.87 to 1.04)	0.94	(0.86 to 1.03)
366-1095 TSDDs	267 (0.5)	938 (0.4)	1.00	(0.87 to 1.15)	0.94	(0.81 to 1.10)
>1095 TSDDs	282 (0.5)	943 (0.4)	1.05	(0.92 to 1.20)	1.05	(0.91 to 1.22)
**Antiarrhythmics**						
Non-use	58720 (99.9)	225402 (99.9)	1.00		1.00	
1-90 TSDDs	9 (0)	37 (0)	0.88	(0.42 to 1.84)	0.73	(0.33 to 1.62)
91-365 TSDDs	7 (0)	20 (0)	1.33	(0.56 to 3.17)	1.23	(0.43 to 3.47)
366-1095 TSDDs	10 (0)	27 (0)	1.17	(0.55 to 2.48)	1.25	(0.57 to 2.71)
>1095 TSDDs	23 (0)	88 (0)	0.98	(0.61 to 1.57)	0.93	(0.56 to 1.54)
**Antimuscarinic bronchodilators**						
Non-use	58764 (100)	225527 (100)	1.00		1.00	
Any use[†]	5 (0)	47 (0)	0.44	(0.17 to 1.11)	0.43	(0.17 to 1.12)

TSDD = total standardised daily dose
[†] Exposure grouped as non-use, any use due to small numbers
[a] Adjusted for other anticholinergic drug types, body mass index, smoking status, alcohol consumption, Townsend deprivation score, ethnic group, coronary heart disease, atrial fibrillation, heart failure, hypertension, hyperlipidaemia, diabetes (type 1 and type 2), stroke, transient ischaemic attack, subarachnoid haemorrhage, renal disease, asthma, chronic obstructive pulmonary disease, anxiety, depression, bipolar disorder, schizophrenia, severe head injury, cognitive decline/memory loss, antihypertensive drugs, aspirin, hypnotics, anxiolytic drugs, NSAIDs, statins and with matching by age, sex, practice and calendar time.

1. Gray SL, Anderson ML, Dublin S, et al. Cumulative use of strong anticholinergics and incident dementia: A prospective cohort study. *JAMA Internal Medicine* 2015;175 (3):401-07.

© 2019 Coupland et al. JAMA Internal Medicine
